# Management of ground tire rubber waste by incorporation into polyurethane-based composite foams

**DOI:** 10.1007/s11356-023-25387-w

**Published:** 2023-01-26

**Authors:** Aleksander Hejna, Paulina Kosmela, Adam Olszewski, Łukasz Zedler, Krzysztof Formela, Katarzyna Skórczewska, Adam Piasecki, Mariusz Marć, Roman Barczewski, Mateusz Barczewski

**Affiliations:** 1 Institute of Materials Technology, Poznan University of Technology, Piotrowo 3, 60-965 Poznań, Poland; 2 Department of Polymer Technology, Gdańsk University of Technology, Narutowicza 11/12, 80-233 Gdańsk, Poland; 3 Department of Molecular Biotechnology and Microbiology, Gdańsk University of Technology, Narutowicza 11/12, 80-233 Gdańsk, Poland; 4 Department of Polymer Technology, Bydgoszcz University of Science and Technology, 85-326 Bydgoszcz, Poland; 5 Institute of Materials Engineering, Poznan University of Technology, Jana Pawła II 24, 60-965 Poznań, Poland; 6 Department of Analytical Chemistry, Gdańsk University of Technology, Narutowicza 11/12, 80-233 Gdańsk, Poland; 7 Institute of Applied Mechanics, Poznan University of Technology, Jana Pawła II 24, 60-965 Poznań, Poland

**Keywords:** Ground tire rubber, Polyurethane foams, Filler modification, Zinc borate, Composites, Volatile organic compounds, Emissions, Gas chromatography

## Abstract

Rapid economic growth implicated the developing multiple industry sectors, including the automotive branch, increasing waste generation since recycling and utilization methods have not been established simultaneously. A very severe threat is the generation of enormous amounts of post-consumer tires considered burdensome waste, e.g., due to the substantial emissions of volatile organic compounds (VOCs). Therefore, it is essential to develop novel, environmentally friendly methods for their utilization, which would hinder their environmental impacts. One of the most promising approaches is shredding, resulting in the generation of ground tire rubber (GTR), which can be introduced into polymeric materials as filler. The presented work is related to the thermomechanical treatment of GTR in a twin-screw extruder with zinc borate, whose incorporation is aimed to enhance shear forces within the extruder barrel. Modified GTR was introduced into flexible polyurethane (PU) foams, and the impact of modification parameters on the cellular structure, static and dynamic mechanical performance, thermal stability, as well as thermal insulation, and acoustic properties was investigated. Emissions of VOCs from applied fillers and prepared composites were monitored and evaluated. Depending on the treatment parameters, beneficial changes in foams’ cellular structure were noted, which enhanced their thermal insulation performance, mechanical strength, and thermal stability. It was proven that the proposed method of GTR thermomechanical treatment assisted by zinc borate particles might benefit the performance of flexible PU foamed composites and hinder VOC emissions, which could broaden the application range of GTR and provide novel ways for its efficient utilization.

**Supplementary Information:**

The online version contains supplementary material available at 10.1007/s11356-023-25387-w.

## Introduction

Due to the rapid development of the automotive sector, the global number of vehicles increased, implicating environmental threats. Except for the most discussed CO_2_ emissions, one of the most severe threats is the generation of vast amounts of waste tires. According to the literature, around one billion tires are withdrawn annually from further global use (Formela 2021). Forecasts indicate that in the following decade, this number will be increased by 20% (Liu et al. 2020). Waste tires are considered dangerous waste with no safe possibility of landfilling, which is often prohibited (Chen et al. 2021). Such a situation is attributed to their high flammability and very harmful consequences of their combustion related to the emissions of hazardous compounds, including sulfur oxides or aromatic hydrocarbons (Nadal et al. 2016). Other threats associated with landfilling tires include retaining rainwater, which may accumulate chemicals from waste tires, creating a pathway for leaching into the natural environment (Wik et al. 2009). Therefore, efficient utilization methods are needed, a goal of many industrial and scientific works. Waste tires can be shredded, leading to the generation of ground tire rubber (GTR), which shows potential for industrial applications. It has been investigated as a concrete component (Thomas and Gupta 2016), asphalt modifier (Sienkiewicz et al. 2017), or in the manufacturing of absorbents (Zedler et al. 2022), but the most extensive area is related to its application as a filler for polymer composites (Ramarad et al. 2015; Fazli and Rodrigue 2020; Phiri et al. 2021). To ensure their satisfactory performance, proper compatibility of GTR with polymer matrix is required (Hejna et al. 2020). Two main strategies for providing compatibility are additional compatibilizer and filler modification applications. In the first case, the additive must show good miscibility with the polymer matrix and simultaneously create interactions with filler particles. Another possibility is the modification of filler particles, particularly their surface, to increase the specific surface area and possibly introduce reactive functional groups, enhancing the interfacial interactions with the matrix (Aoudia et al. 2017).

GTR modifications are mainly realized by grafting chemical compounds on the particles’ surface or their partial devulcanization and oxidation, which increases the surface area and enhances the GTR reactivity (Zedler et al. 2019b). Devulcanization results in partial degradation of rubber 3D network and can be realized using mechanical, thermomechanical, chemical, ultrasonic, microwave, and other methods (Seghar et al. 2019). One of the most promising and environmentally beneficial methods is reactive extrusion, which could be performed continuously, without solvents, with reduced heat demand, and using the equipment commonly available in polymer technology (Zedler et al. 2019a). Moreover, it enables the application of additional GTR modifiers. Nevertheless, the partial decomposition of GTR due to the substantial shear forces and elevated temperatures acting on the material during thermomechanical treatment, except for the above-mentioned benefits, shows some drawbacks. The most serious is associated with the emissions of volatile organic compounds (VOCs) during modification, as well as from the final product—devulcanized GTR (Gągol et al. 2015). The emissions may be attributed to the evaporation of additives applied during rubber manufacturing, like plasticizers or curing agents or their degradation products. Moreover, the decomposition of macromolecular chains of natural and synthetic rubbers may lead to the formation of volatile aliphatic and aromatic hydrocarbons, as well as aldehydes and ketones, often considered harmful to human health and the environment (Formela 2022). Therefore, it is essential to develop such applications for modified GTR, which would hinder harmful emissions. Incorporating it into polymer composites may provide such an effect in the case of the proper compatibility, beneficially including covalent bonding at the interface. Such an effect can be achieved by applying reactive compatibilizers or incorporating GTR into matrices containing proper functional groups (Aoudia et al. 2017). Among the potential candidates, polyurethanes (PU) seem auspicious due to the presence of highly reactive isocyanates in the reaction system, which could react with GTR functional groups generated during modifications (Hejna et al. 2016). As mentioned above, to provide sufficient interface strength, the GTR surface should be well developed, which, however, shows an inferior impact on VOC emissions. Therefore, during the developing of polymer/GTR composites with reduced environmental impacts, it is crucial to seek the balance between strong interfacial adhesion resulting from the surface development and oxidation of rubber particles and simultaneously intensified VOC emissions.

The presented study aimed to investigate the thermomechanical modification of GTR with zinc borate in the twin-screw extrusion process as a novel, highly effective treatment method and further incorporation of modified GTR in a flexible, foamed polyurethane matrix. The goal of zinc borate introduction was to provide additional friction during the thermomechanical modification of GTR, which could enhance the specific surface area and reduce GTR particle size. These two factors are often crucial for the performance of polymer composites (Fu et al. 2008). Moreover, additions of inorganic modifiers may noticeably enhance the thermal stability of polymer materials (Morgan and Putthanarat 2011). The zinc borate was used in the amount of 40 parts by weight with respect to the GTR, and the compositions were extruded at varying temperatures and screw speeds. The structural changes in GTR/zinc borate compositions were correlated with VOC emissions. Then, compositions were introduced into foamed polyurethane matrix in the fixed amount of 15 parts by weight (pbw). The VOC emission profiles of composites were analyzed and used to evaluate the interfacial compatibility and its impact on the cellular structure, insulation, mechanical, and thermal performance.

## Experimental

### Materials

The materials used in the presented study are listed in Table 1.

**Table 1:** List of materials used in the presented study

Material	Producer	Properties/additional information
PTMG 2000	Sigma Aldrich (USA)	Poly(tetramethylene ether)glycol, molar mass—2000 g/mol
Glycerol	Sigma Aldrich (USA)	Hydroxyl value—1800 mg KOH/g
Toluene diisocyanate (TDI)	Sigma Aldrich (USA)	Mixture of 2,4-TDI and 2,6-TDI in the 80/20 ratio
Dabco33LV (DABCO)	Air Products (USA)	Catalyst, 33 wt% solution of 1,4-diazabicyclo[2.2.2]octane in dipropylene glycol
Dibutyltin dilaurate (DBTL)	Sigma Aldrich (USA)	Organic tin catalyst
Tegostab B8460	Evonik Industries AG (Germany)	Foam stabilizer, polyether polydimethylsiloxane copolymer
Distilled water	–	Chemical blowing agent
Ground tire rubber	Grupa Recykl S.A. (Poland)	Filler, mean particle size = 0.6 mm
Firebrake® 500	U.S. Borax (USA)	Anhydrous zinc borate, median particle size 9 μm

### Modification of ground tire rubber with zinc borate

Thermomechanical modification of GTR with zinc borate was performed in a co-rotating twin-screw extruder EHP 2×20 Sline from Zamak Mercator (Poland). GTR was introduced into the extruder barrel with 40 parts by weight of zinc borate. To investigate the impact of processing parameters, the barrel temperature was set at 150 or 200 °C, while the screw speed was 80, 150, or 350 rpm. For comparison, a premix without thermomechanical treatment was also prepared.

### Preparation of composite foams

Polyurethane foams were prepared on a laboratory scale by a two-step method. The first step was the preparation of the prepolymer. Poly(tetramethylene ether)glycol (PTMG) was mixed with a calculated amount of toluene diisocyanate (TDI), and synthesis was carried out at 60 °C under a vacuum for 120 minutes. The content of unreacted isocyanate groups in prepolymer was 18.20%. Next, the blend, including PTMG, glycerol, catalysts, foam stabilizer, blowing agent, and, in the case of composite foams, ground tire rubber, was prepared. In the case of composite foams, GTR was homogenized with PTMG and glycerol for 30 seconds prior to incorporating other components. When the complete blend was prepared, it was mechanically mixed with a previously prepared prepolymer for 10 seconds and poured into a mold heated up to 60 °C. After demolding, samples were held at 60 °C for 24 h. Table 2 contains the details of foam formulations, including parameters of GTR treatment.

**Table 2:** Formulations of prepared polyurethane composite foams

Component	Foam symbol						
	1	2	3	4	5	6	7
	Content (pbw)						
PTMG	63.86						
Glycerol	5.11						
DABCO	0.31						
DBTL	0.27						
B8460	0.20						
Water	0.20						
TDI	30.06						
GTR	15.00						
GTR treatment parameters	Premix	150°C/80 rpm	200°C/80 rpm	150°C/150 rpm	200°C/150 rpm	150°C/350 rpm	200°C/350 rpm

### Characterization techniques

Particle size distribution of GTR/zinc borate compositions was determined using a laser particle sizer Fritsch ANALYSETTE 22 apparatus (Germany) operated in the range of 0.08–2000 μm.

The microstructure of applied GTR compositions and resulting composites was evaluated using a scanning electron microscope (SEM) MIRA3—produced by Tescan (Czech Republic). The thin carbon coating with a thickness of approximately 20 nm was deposited on samples using Jeol JEE 4B vacuum evaporator from Jeol USA (USA). The cellular structures of foams were analyzed using an accelerating voltage of 5 kV. The secondary electron detector was used. Obtained images were analyzed with ImageJ software.

To assess the emissions of selected aromatic hydrocarbons released to the gaseous phase from the investigated materials, the Markes’ Micro-Chamber/Thermal Extractor™–μ-CTE™ 250 (Markes International, Ltd.) was employed. This device (containing 4 equally stainless steel chambers—114 cm^3^ internal volume each) gives a possibility to perform the emission investigations under controlled seasoning conditions, considering temperature (ranging from room temp. up to 250 °C) and air exchange rate (defined by the inert gas flow rate in the range from 10 mL/min up to 500 mL/min). Information about the design and potential working parameters of the μ-CTE™ 250 system was described in detail elsewhere (Schripp et al. 2007; Marć et al. 2017).

Samples of analytes, which were emitted to the gaseous phase from the investigated materials placed inside mentioned chambers, were collected using Tenax TA stainless steel tubes (60/80 mesh, preconditioned, Merck KGaA, Darmstadt, Germany).

Retained on a Tenax TA, samples of organic compounds were extracted using the two-stage thermal desorption (TD) technique. To carry out this process, the following TD systems were applied: (i) Markes Series 2 Thermal Desorption Systems; UNITY/TD-100 (Markes International, Inc.) attached by heating transfer line to gas chromatography-flame ionization detector (GC-FID); (ii) Markes Unity v.2, (Markes International, Inc.) linked by heating transfer line to GC with a mass spectrometer (MS). The qualitative and quantitative analysis of investigated samples was performed using GC-FID (Agilent Technologies 7820A GC; capillary column: J&W DB-1, USA; Data processing system: OpenLAB CDS ChemStation). The main representatives of aromatic hydrocarbons emitted from the investigated samples were determined using a certified reference solution—13 representatives of VOCs in 1 mL of MeOH at a concentration level of 2000 μg/mL each (VOC EPA Mix 2, Supelco, Bellefonte, PA, USA). Identification of representatives of aromatic hydrocarbons using the TD-GC-FID system was carried out by comparison of the retention times obtained for investigated samples and employed certified reference solution. The quantification of the representatives of aromatic hydrocarbons was carried out using external standard technique (ESTD-VOC EPA Mix 2). The five-point calibration solutions in 1 mL MeOH were prepared—analytes range from 2 ng/μL up to 500 ng/μL. The calibration protocol was described in detail elsewhere (Zabiegala et al. 2010; Zabiegała et al. 2011; Marć et al. 2014). The limit of detection (LOD) was assessed as a signal-to-noise (S/N) ratio, and the average value was 0.33 ng. In addition, to expand the identification potential of performed investigations, the GC-MS (GC Agilent Technologies 6890; 5873 Network Mass Selective Detector, Agilent Technologies; capillary column J&W HP-1ms, USA; Data processing system: ChemStation) was used. The screening identification of emitted organic compounds was performed with the use of the mass spectra database (NIST 2.0 Mass Spectral Library, The NIST Mass Spectral Search Program for the NIST/EPA/NIH Mass Spectral Library Version 2.0d, USA). Only relationships with a probability higher than 85% agreement were considered. Detailed information about the steps of analytical protocol applied to assess the type and amount of organic compounds emitted to the gaseous phase from the samples of investigated materials is shown in Supplementary Figure 1 and partially described elsewhere (Wiśniewska et al. 2022b; Marć et al. 2022).

After conditioning, foamed polyurethane composites were cut into samples whose properties were later determined following the standard procedures.

Color coordinates (L*, a*, and b*) in the CIELab space were determined for the samples using an NR145 colorimeter from Envi Sense (Poland) operating in a 45°/0° geometry. The details on the calculation of other color parameters like total color difference (ΔE*), chroma, or hue, as well as on the conversion of CIELab color coordinates to the commonly used Adobe RGB color space, have been provided in previous work (Hejna et al. 2021b).

The content of open cells in foamed composites was determined using Ultrapyc 5000 Foam gas pycnometer from Anton Paar (Austria). The following measurement settings were applied: gas—nitrogen; target pressure—3.0 psi; measurement type—corrected; flow direction—sample first; target temperature—20.0 °C; flow mode—monolith; cell size—medium, 45 cm^3^; preparation mode—flow; time of the gas flow—0.5 min.

The thermal conductivity coefficient (*λ*) or prepared polyurethane foams was determined using the heat flow meter HFM 446 from Netzsch (Germany). Samples with a thickness of 4 cm were tested in the temperature range from 1 to 19 °C using the average temperature of 10 °C.

The tensile strength of foams was estimated following ISO 1798. The beam-shaped samples with 10 × 10 × 100 mm^3^ dimensions were measured with a slide caliper with an accuracy of 0.1 mm. The tensile test was performed on a Zwick/Roell tensile tester (Germany) at a 500 mm/min constant speed.

Dynamical mechanical analysis (DMA) was performed using a Q800 DMA instrument from TA Instruments (USA) at a heating rate of 4 °C/min and the temperature range from −100 to 150 °C. Samples were cylindrical-shaped, with dimensions of 10 × 12 mm.

The thermogravimetric (TGA) analysis of composites was performed using the TG 209 F3 apparatus from Netzsch (Germany). Samples weighing approx. 10 mg were placed in a ceramic dish. The study was conducted in a nitrogen atmosphere in the range of 30–800 °C with a temperature increase rate of 10 °C/min.

The determination of the sound absorption coefficients of material samples was carried out in accordance with the ISO 10534-2 and ASTM E1050-8 standard. The following equipment was used to carry out the tests: two BSWA impedance tubes (SW422 and SW 744), MC 3242 data acquisition hardware, PA50 power amplifier, BSWA VA LAB4 software (BSWA-Technology Co. Ltd., China), and two MI 19 microphones—¼ inch IEPE standard (Roga Instruments, Germany). The measuring system was calibrated with a CA114 acoustic calibrator (BSWA Technologies Co. Ltd. China). Atmospheric pressure, temperature, and air humidity were monitored with the LB-575 climate meter (produced by LAB-EL, Poland). The preparation of samples for testing included cutting out from the base material (approximately 22 mm thick) and discs with 30 and 100 mm diameters. The samples were tested, from which about 6 mm of the uneven upper layer was removed. This treatment was also aimed at exposing the internal cellular structure of the material (foam). After cutting off the top layer, the samples were 16 mm thick. The test results are presented as characteristics containing the values of sound absorption coefficients in 1/3 octave bands (100–6300 Hz). The characteristics were created based on partial results obtained in the bands 63–500 Hz and 250–1600 Hz (using an impedance tube with a diameter of 100 mm and different spacing of microphones) and in the band 1000–6300 Hz (using a tube SW744 with a diameter of 30 mm). The final result for each sample is the result of averaging three measurements. In addition, the average value of the sound absorption coefficient (*α*_avg_) was determined for each of the types of material following Eq. (1):

1 $$\alpha_\text{avg}=\frac1n\sum\limits_{i=1}^n\alpha_{f(i)}$$

where *α*_*f*_(*i*) is the sound absorption coefficients (at the center frequencies *f*(*i*) from 100 Hz to 6.3 kHz) and *n* is the number of 1/3 octave bands.

## Results and discussion

### Properties of modified ground tire rubber

**Fig. 1:**
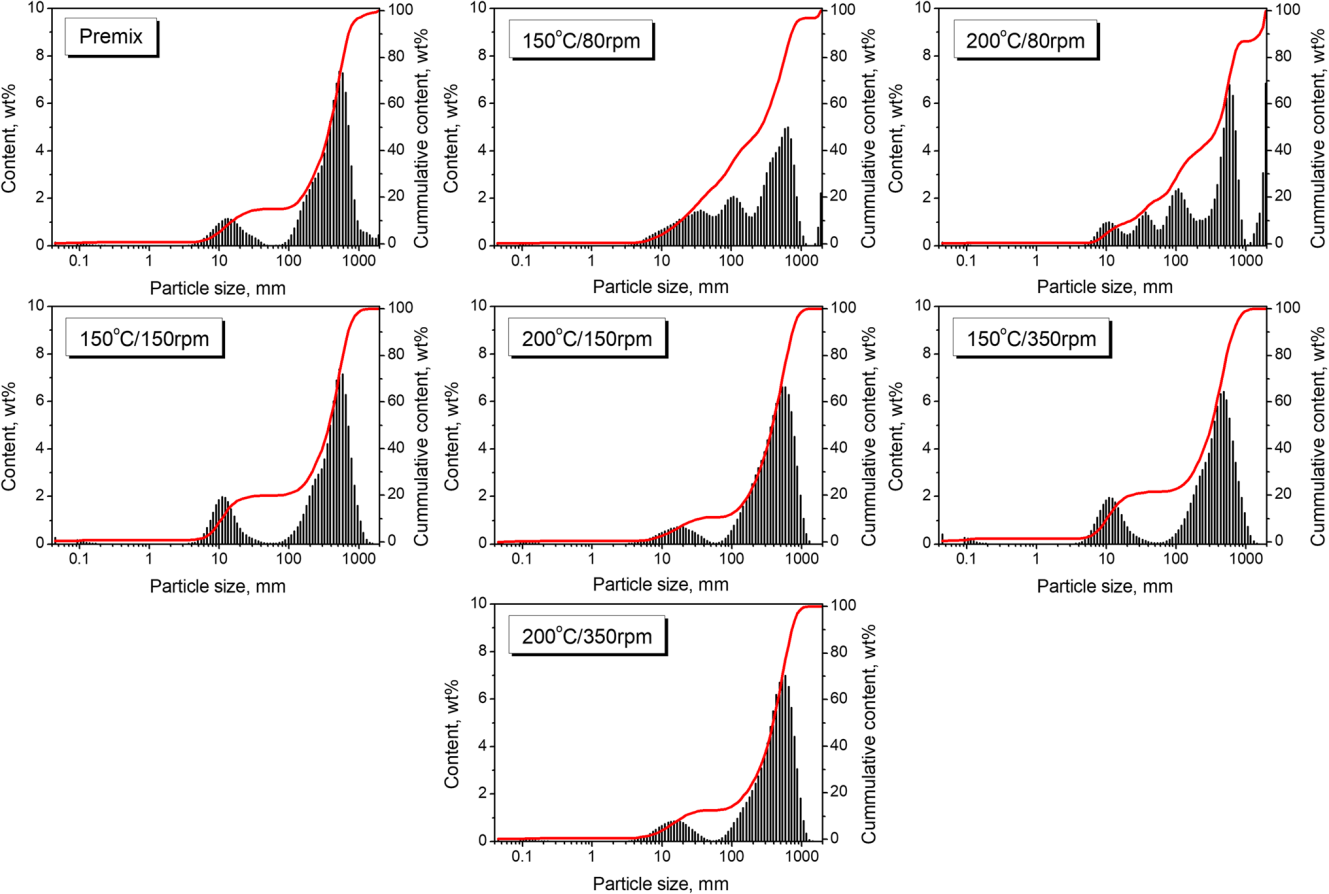
Particle size distribution of GTR/zinc borate compositions thermomechanically modified under different conditions

The size of filler particles and often associated specific surface area significantly impact composite materials’ performance. In the case of polyurethane foams, this impact is even more substantial because it affects not only the mechanical properties but also the course and rate of the foaming process, as well as the cellular structure, which is critical, e.g., for thermal insulation or sound absorption performance (Li et al. 2014; Sung and Kim 2017). Therefore, Fig. 1 presents the changes in the particle size distribution of GTR/zinc borate compositions resulting from thermomechanical treatment in a twin-screw extruder. Initially, the composition was heterogeneous and consisted of two types of particles with noticeably different sizes in the range of 5–45 μm and 100–1500 μm, respectively. Depending on the applied parameters, extrusion affected the particle size distribution, attributed to the shear forces acting on the material and the friction between particles, which may cause their grinding and coalescence. The most significant effects were noted for the lowest screw speed, which resulted in longer materials’ residence time and a higher degree of filling of the extruder, which increased the shear forces acting between the GTR and zinc borate particles (Suparno et al. 2011). As a result, part of the inorganic particles was compressed onto the surface of GTR, which was also partially ground, as indicated by the particle size distribution curves and presented in Fig. 1. For higher values of screw speed, the effect was not so significant, which can be attributed to the shorter residence time and lower degree of filling of the extruder, which reduced the interparticle shearing (Zhang et al. 2008). Irrespective of the screw speed, increasing the temperature of the extruder barrel increased the content of bigger particles, which can be seen in cumulative curves. Such an effect can be associated with an enhanced agglomeration of particles, as suggested by Fig. 1.

**Fig. 2:**
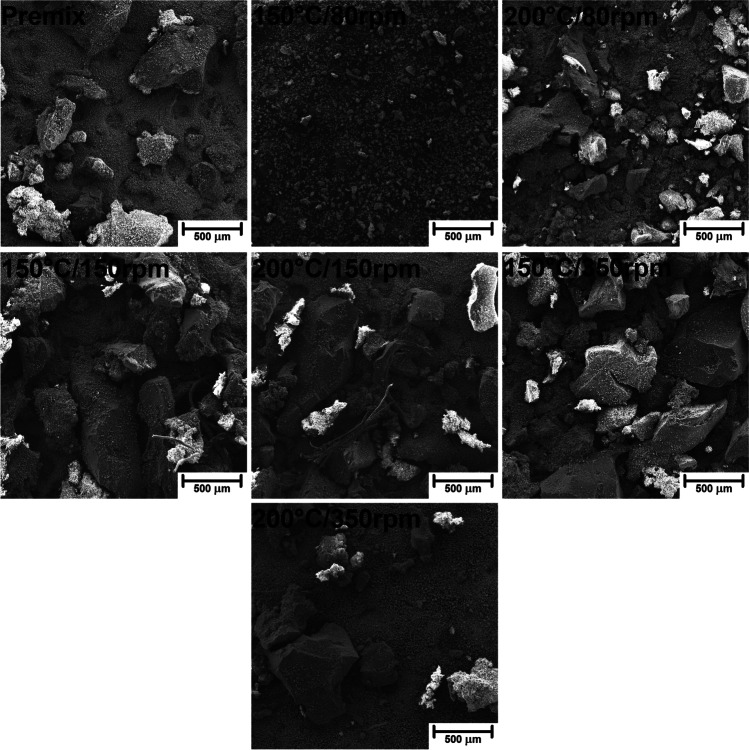
Images of GTR/zinc borate compositions obtained with SEM microscopy at a magnification of 100×

The differences in the particle size distribution of GTR/zinc borate compositions resulting from thermomechanical treatment can also be seen in Fig. 2, showing images obtained with SEM microscopy. Clearly, for higher values of screw speed, the particle size of GTR is relatively similar to the initial one observed for premix. More significant variations were noted for compositions modified at 80 rpm, confirming the data resulting from particle size measurements. As mentioned above, such an effect could be associated with a longer residence time of material in the extruder barrel caused by lower screw speed and stronger interparticle shearing induced by a higher degree of filling of the extruder.

**Fig. 3:**
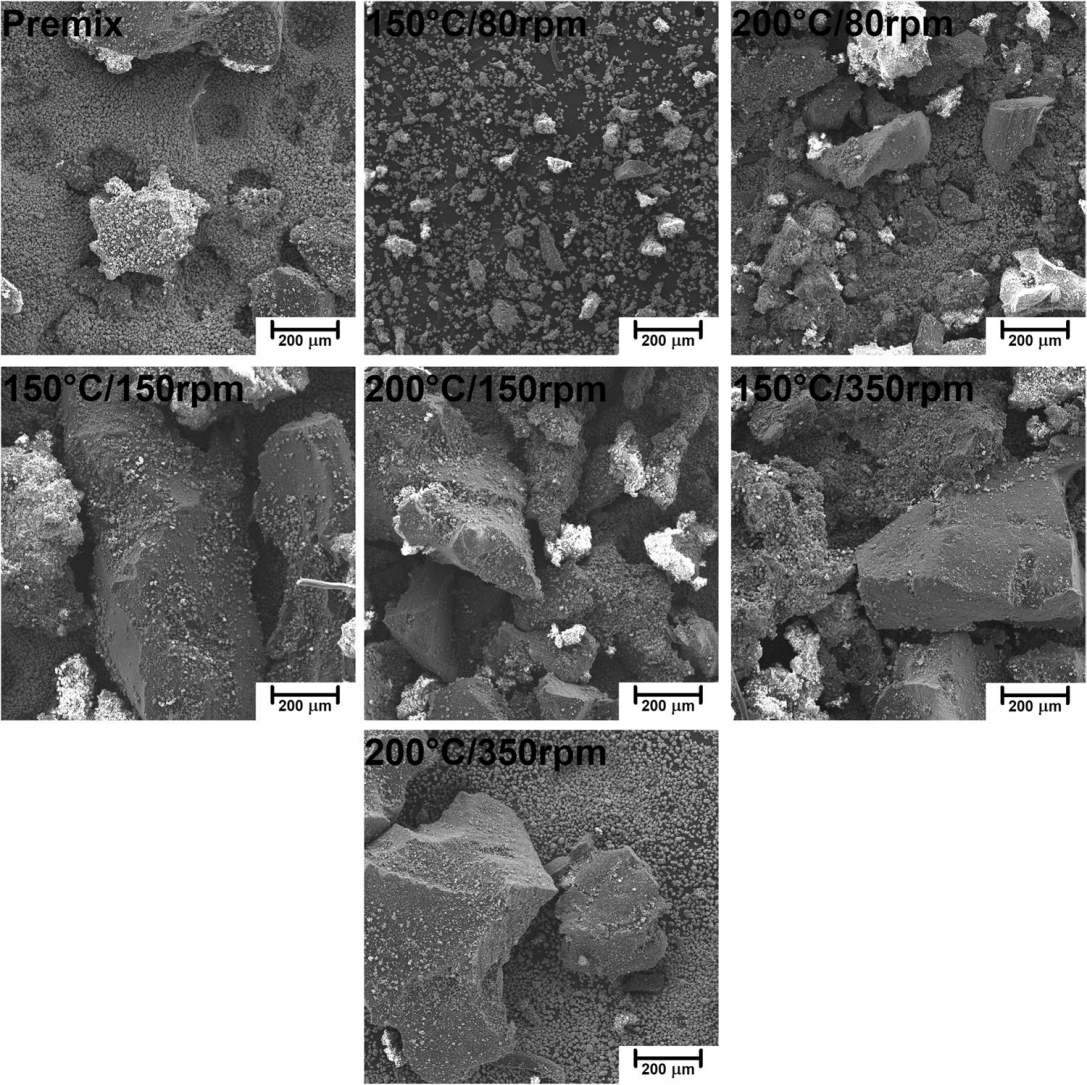
Images of GTR/zinc borate compositions obtained with SEM microscopy at a magnification of 200×

Except for the information related to particle size, SEM images may provide insights into the structure of particles’ surface, which is crucial for the performance of composite materials. Figure 3 shows the images of GTR/zinc borate compositions at higher magnification, indicating the difference in surface development of GTR particles between particular samples. Again, the differences between premix and compositions thermomechanically modified at 150 and 350 rpm are relatively small. It can be seen that particles of zinc borate are loosely deposited on the surface of GTR particles, with the only minor share of particles compressed into the surface. The application of lower screw speed noticeably affected the surface roughness of GTR particles.

**Fig. 4:**
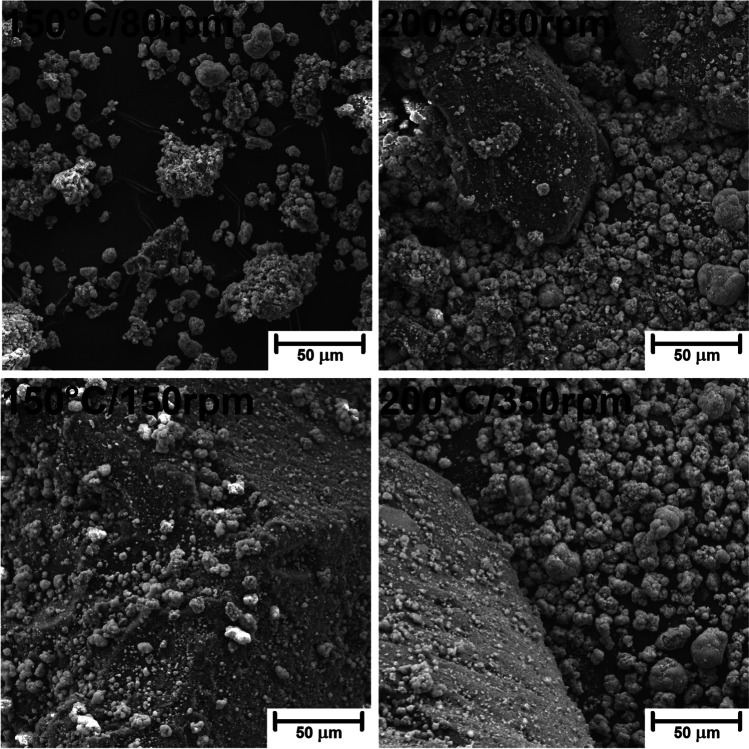
Images of selected GTR/zinc borate compositions obtained with SEM microscopy at a magnification of 1000×

For more detailed analysis, in Fig. 4, samples 150°C/80rpm and 200°C/80rpm have been additionally presented at a higher magnification of 1000**×** and compared with 150°C/150rpm and 200°C/350rpm samples. It can be seen that GTR particles modified at 150 °C and 80 rpm are characterized by significantly the roughest surface, which can be ascribed to the abrasion of the surface with zinc borate particles (also inducing particle size reduction) and pressing inorganic particles into rubber surface. A similar phenomenon, but to a lower extent, can be noted for composition 200°C/80 rpm, pointing to the agglomeration of GTR particles and reduced interparticle shearing related to the partial GTR decomposition. In the case of samples modified at higher screw speed, two components of compositions can be distinguished more easily because zinc borate particles are rather freely deposited on the GTR surface. For sample 150°C/150 rpm, only a minor portion of inorganic particles is pressed into the rubber surface. As a result, the surface of GTR particles is noticeably smoother.

**Fig. 5:**
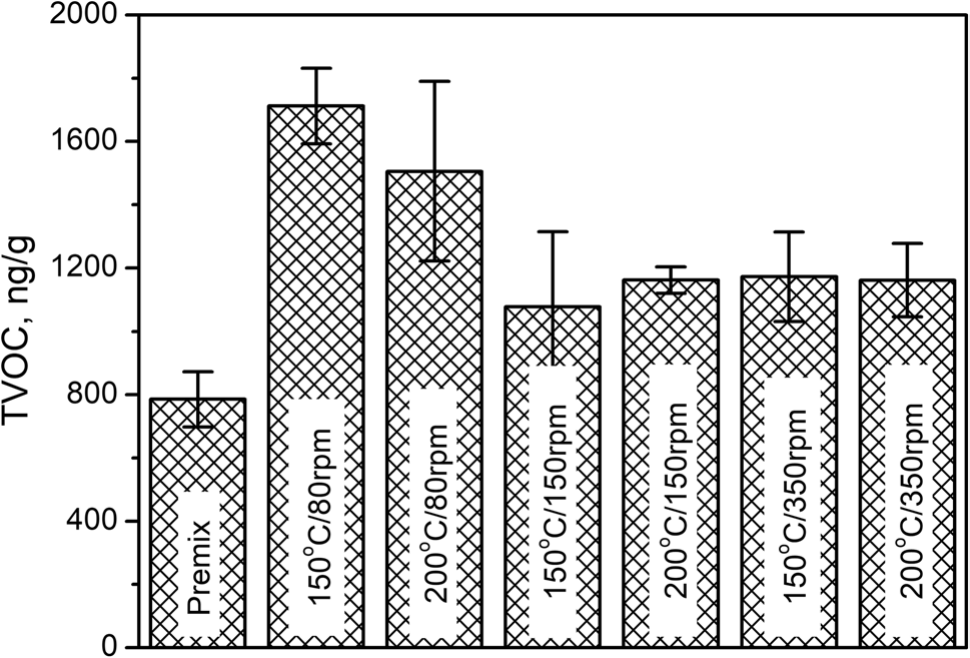
Total amount of VOCs emitted from thermomechanically modified GTR/zinc borate compositions

As mentioned above, an important issue related to the management of GTR is the emission of VOCs, among which can be found compounds harmful to humans and the environment (Gągol et al. 2015; Zedler et al. 2020a). This aspect is not only associated with the chemical composition of GTR but also with their physical characteristics like particle size or surface development and may be influenced by various treatments aimed at developing GTR management strategies. Therefore, Fig. 5 presents the total amount of emitted VOCs (TVOC) from applied GTR/zinc borate compositions. The lowest TVOC value was noted for a premix of rubber particles with zinc borate applied without thermomechanical treatment. Such an effect could be attributed to the relatively smooth surface of unmodified rubber particles, as shown in Fig. 3. Moreover, without treatment, GTR particles have only been mixed with zinc borate, so the crosslinked rubber structure was unaffected. After thermomechanical treatment, depending on the applied parameters, the surface of particles was roughened, but also, due to the shear forces acting on the material in the extruder barrel, GTR was partially devulcanized (Wiśniewska et al. 2022a). Such an effect generates lower-molecular weight compounds, which due to their vapor pressure, can be classified as VOCs (Formela 2022). Moreover, the loosening of crosslinked structures facilitates the migration of volatile compounds originating from additives or processing agents applied during the manufacturing of tires (Saputra et al. 2021). The highest TVOC values were noted for the lowest screw speed of 80 rpm, which can be associated with the highest surface development and roughness, as well as the greatest extent of structure devulcanization. As mentioned above, these effects are induced by the longest residence time of the material in the extruder and the highest degree of filling of the extruder, which enhances interparticle shearing. For higher values of screw speed, the TVOC parameter was hardly affected. Its values were similar, considering standard deviation values. Such an effect confirms the lower extent of changes caused by thermomechanical treatment of GTR/zinc borate compositions suggested by the particle size distribution curves and SEM images presented above.

Table 3 presents the data on the VOCs emitted from GTR/zinc borate compositions along with information on their potential origin. Clearly, by far, the most numerous group of detected compounds is aromatics, whose presence is mostly attributed to the decomposition of styrene-butadiene rubber (SBR) used in tire manufacturing (Formela 2022). The presence of aliphatic hydrocarbons and terpenes can be attributed to the degradation of natural rubber (NR) (Kamarulzaman et al. 2019; Marć et al. 2021). Hydrocarbons are generated during the decomposition of long polyisoprene chains, while the presence of terpenes can be attributed to the plant origin of natural rubber. Moreover, among the detected VOCs can be found aldehydes and ketones of various origins, either generated during NR or SBR decomposition, but also applied in the production of rubber additives, like antioxidants (Allen et al. 2000; Sakai et al. 2022). The presence of rubber additives may also yield emissions of benzothiazole applied as vulcanizing agent or methyl cumyl ether resulting from the decomposition of peroxides, but also dimerization of α-methylstyrene used as a chain transfer agent in SBR production (Ghosh and Chaudhuri 2006; Kamarulzaman et al. 2019).

**Table 3:**
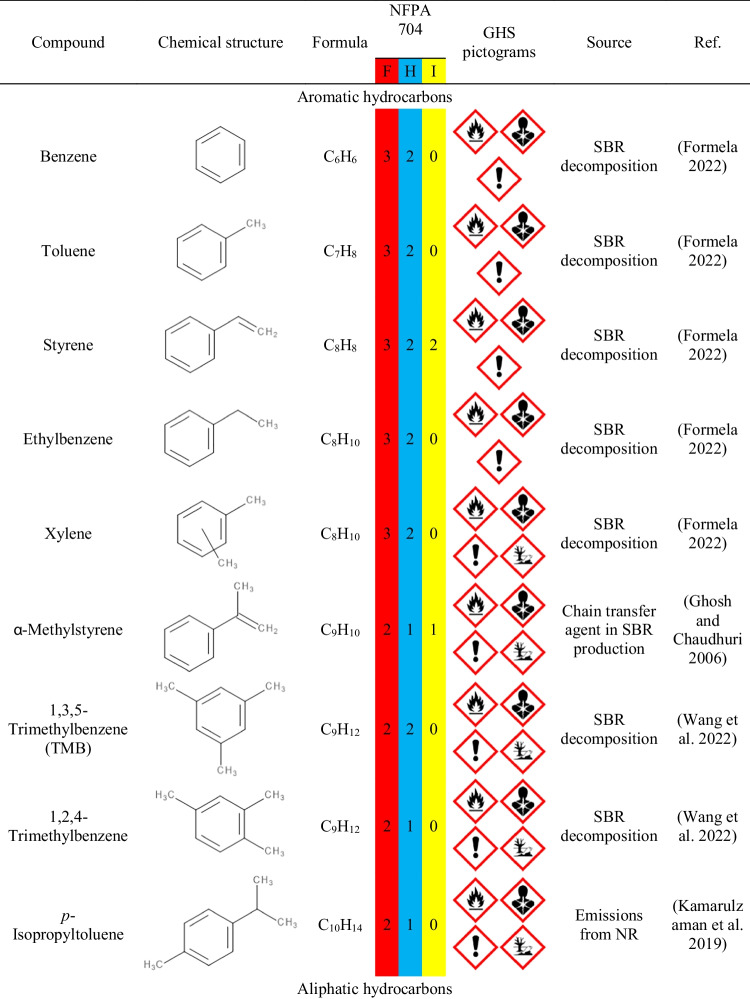

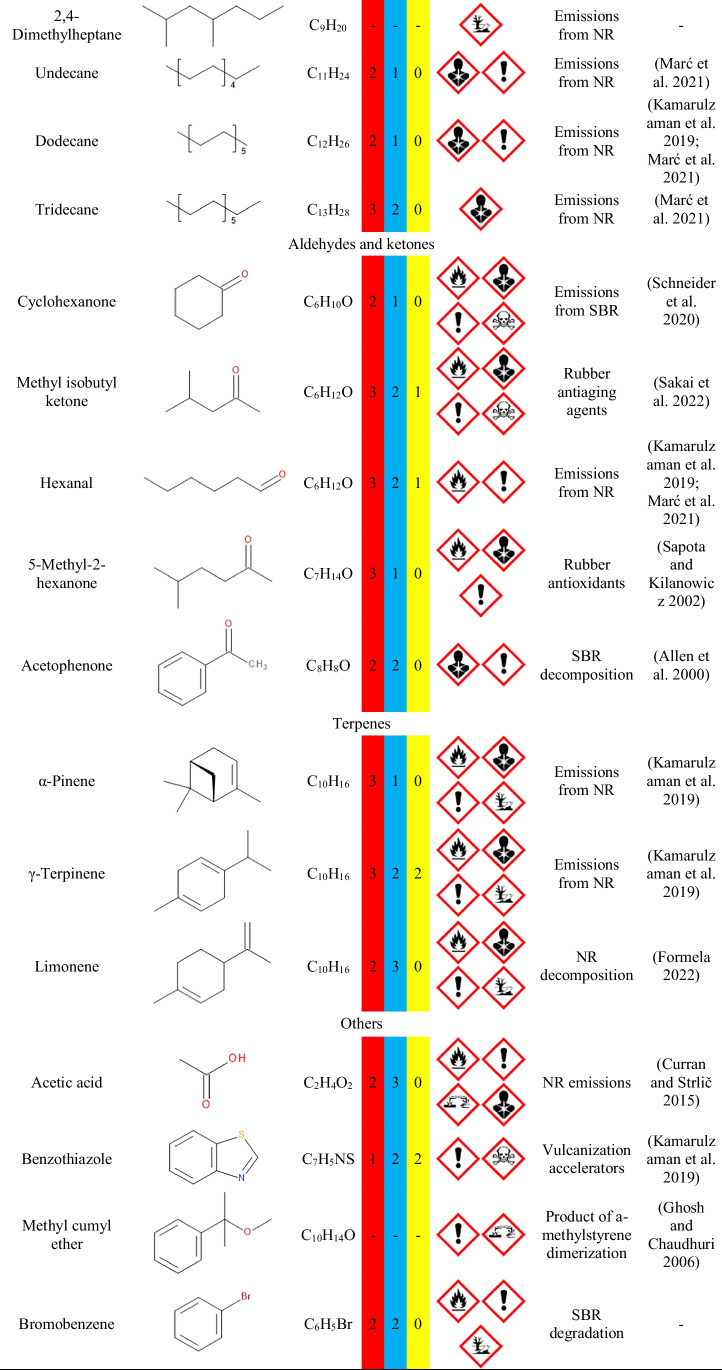
List of VOCs emitted from GTR/zinc borate compositions along with their origin and information on occupational safety and health hazards

Moreover, Table 3 provides information on occupational safety and health hazards of detected VOCs according to the international safety standards: Globally Harmonized System of Classification and Labelling of Chemicals (GHS) and the NFPA 704: Standard System for the Identification of the Hazards of Materials for Emergency Response (Hejna et al. 2021c). It can be seen that the majority of detected VOCs pose significant threats owing to their flammability and potential health hazards. Some of them are also considered harmful to the environment. Presented data confirm the results presented in other works indicating the hazardous character of waste tires and pointing to the need to reduce VOC emissions from GTR (Fazli and Rodrigue 2020; Wiśniewska et al. 2021).

**Fig. 6:**
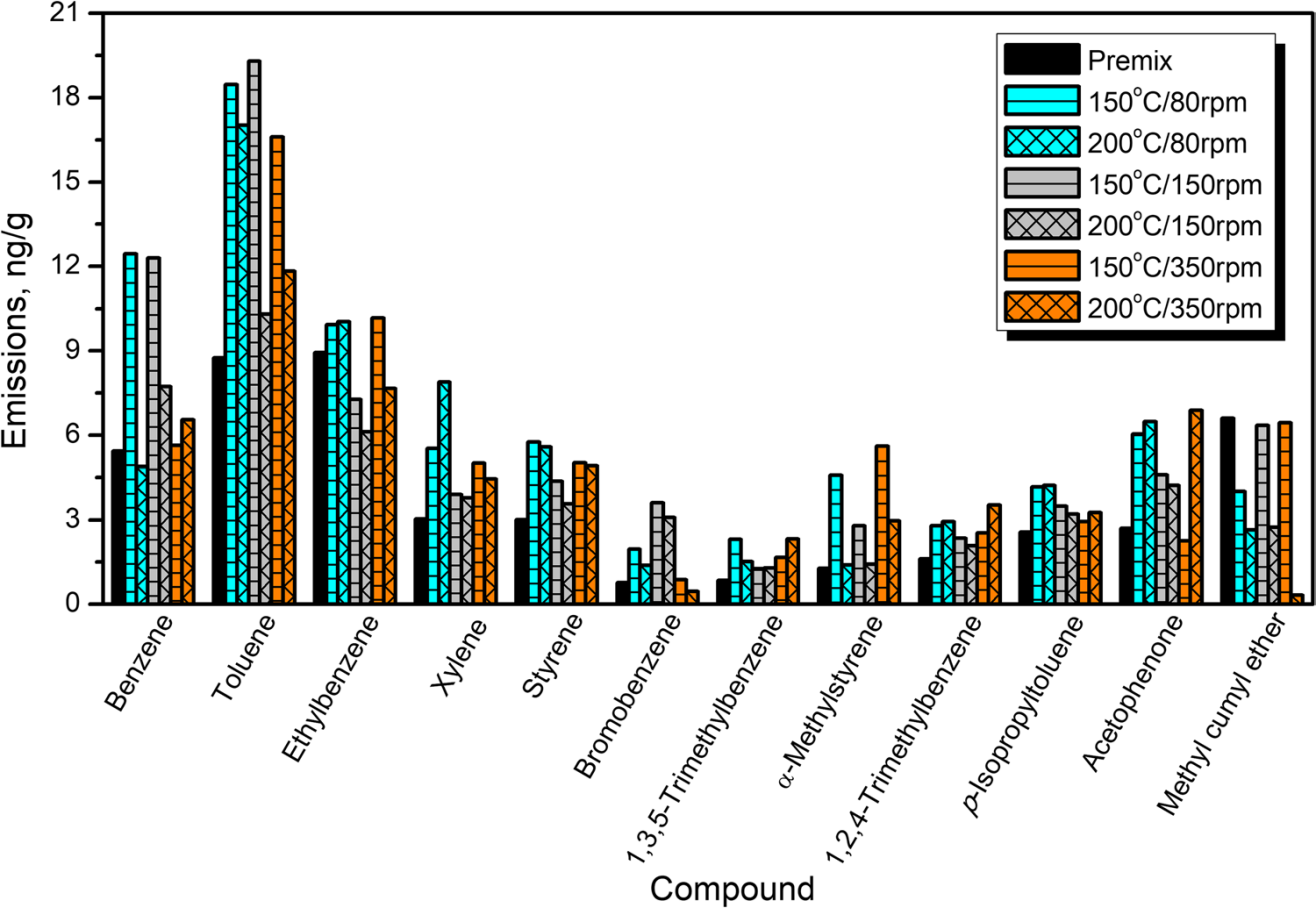
The detailed emissions of aromatic compounds from GTR/zinc borate compositions based on the applied certified reference material

Figure 6 shows the detailed emissions of aromatic compounds based on the applied certified reference material. Aromatic hydrocarbons have been selected for more detailed investigation due to their major contribution to the overall VOC emissions in recycled rubber materials (Li et al. 2019; Huang et al. 2022). According to the literature data (Wang et al. 2022), aromatics account for over 64% of VOC emissions during GTR devulcanization, which partially occurs during performed thermomechanical treatment. In the presented case, the most abundant aromatics emitted were benzene, toluene, ethylbenzene, xylenes, and styrene. Their presence can be associated with their common use in the rubber industry, e.g., as solvents and their generation during thermomechanically induced partial decomposition of GTR.

For a deeper analysis of the potential interactions between the compounds emitted from GTR/zinc borate compositions, the Pearson correlation coefficients (PCC) were determined. Their values calculated for the above-mentioned aromatic compounds and TVOC parameters are presented in Table 4. The bolded PCC values point to a strong (between 0.6 and 0.8) or very strong (between 0.8 and 1.0) correlation between particular compounds (Marć 2020). The strongest correlations were noted for emissions of 1,3,5-trimethylbenzene, 1,2,4-trimethylbenzene, acetophenone, and TVOC parameter. The presence of trimethylbenzenes among the VOCs emitted from rubber materials has been repeatedly reported in multiple works (Yu and Crump 1998; Juntarachat et al. 2013; Kamarulzaman et al. 2019; Sakai et al. 2022). The significant contribution of acetophenone to the TVOC parameter can be associated with the composition of applied GTR, in particular, the presence of styrene-butadiene rubber (SBR). Similar to natural rubber, SBR is vulnerable to oxidation due to the presence of double bonds in the butadiene phase (Rezig et al. 2020). During the oxidation of the polystyrene phase, which occurs during thermomechanical treatment of GTR, acetophenone groups are formed (Allen et al. 2000). Acetophenone was also noted among the VOCs emitted from GTR in other works (Wiśniewska et al. 2021, 2022b). Among the particular aromatic compounds, strong correlations were noted between emissions of toluene, ethylbenzene, xylenes, and styrene, all of which have been reported as products of SBR rubber oxidative or thermal degradation (Kwon and Castaldi 2008; Formela 2022).

**Table 4:** Pearson correlation coefficients for emissions of aromatic compounds from GTR/zinc borate compositions

	1	2	3	4	5	6	7	8	9	10	11	12	13
1: TVOC	1.00	0.58	0.36	0.13	0.57	0.36	0.23	**0.87**	0.22	**0.92**	**0.64**	**0.85**	**−0.72**
2: benzene		1.00	0.25	**−**0.41	**−**0.20	**−**0.14	**0.72**	0.52	0.16	0.57	0.20	0.57	**−**0.25
3: toluene			1.00	0.42	0.55	**0.83**	0.15	0.54	0.59	0.38	**0.70**	0.30	0.12
4: ethylbenzene				1.00	0.59	**0.69**	**−0.82**	0.34	0.49	0.09	0.31	**−**0.08	0.26
5: xylene					1.00	**0.77**	**−**0.26	0.48	0.11	0.55	**0.79**	0.55	**−**0.47
6: styrene						1.00	**−**0.24	0.58	0.55	0.25	**0.79**	0.23	**−**0.04
7: bromobenzene							1.00	0.08	**−**0.21	0.25	0.18	0.43	**−**0.33
8: 1,3,5-TMB								1.00	**0.63**	**0.73**	**0.60**	0.55	**−**0.40
9: α-methylstyrene									1.00	0.21	0.10	**−**0.23	0.37
10: 1,2,4-TMB										1.00	0.47	**0.78**	**−0.61**
11: p-isopropyltoluene											1.00	**0.73**	**−**0.50
12: acetophenone												1.00	**−0.79**
13: methyl cumyl ether													1.00

### Properties of polyurethane/ground tire rubber composite foams

**Fig. 7:**
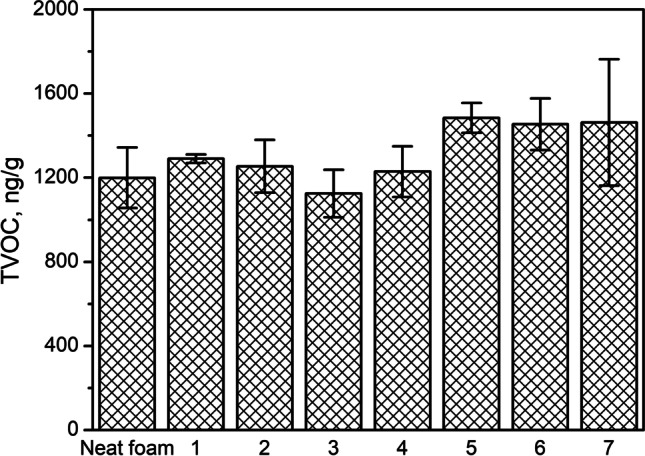
Total amount of VOCs emitted from prepared PU/GTR composites and unfilled PU foam

Figure 7 shows the values of the TVOC parameter for prepared composites. Moreover, for comparison, the value for neat, unfilled foam obtained with a similar formulation but without the addition of GTR/zinc borate composition was provided. It can be seen that the TVOC parameter for neat foam equals 1200 ng/g, while for composite samples it is in the range of 1125–1485 ng/g. No straightforward correlation between TVOC values for fillers and composites can be noted, which points to the complexity of the VOC emission mechanism in the case of composite materials. The introduction of GTR/zinc borate premix without additional treatment caused a slight increase in TVOC parameters compared to unfilled foam. The only minor increase can be attributed to the low value of TVOC for premix, as presented in Fig. 5. Composites containing fillers modified at higher screw speeds (150 and 350 rpm) are generally characterized by higher TVOC values.

On the other hand, the incorporation of GTR/zinc borate compositions treated at 80 rpm reduced VOC emissions despite the significantly higher TVOC values of fillers. It can be associated with the surface development of GTR particles and potentially its partial oxidation. It suggests that except for the extent of the emissions from fillers and unfilled foam, the compatibility of composite and interfacial adhesion plays a crucial role. Surface development of GTR particles during thermomechanical treatment facilitates VOC emissions from filler but simultaneously may provide additional interactions with the PU matrix, including covalent bonding between isocyanates and hydroxyl groups present on the surface of rubber particles. It leads to the strengthening of interfacial interactions with the PU matrix and better encapsulation of GTR particles by the PU matrix, hindering VOC emissions from the final composite (Li et al. 2021).

**Fig. 8:**
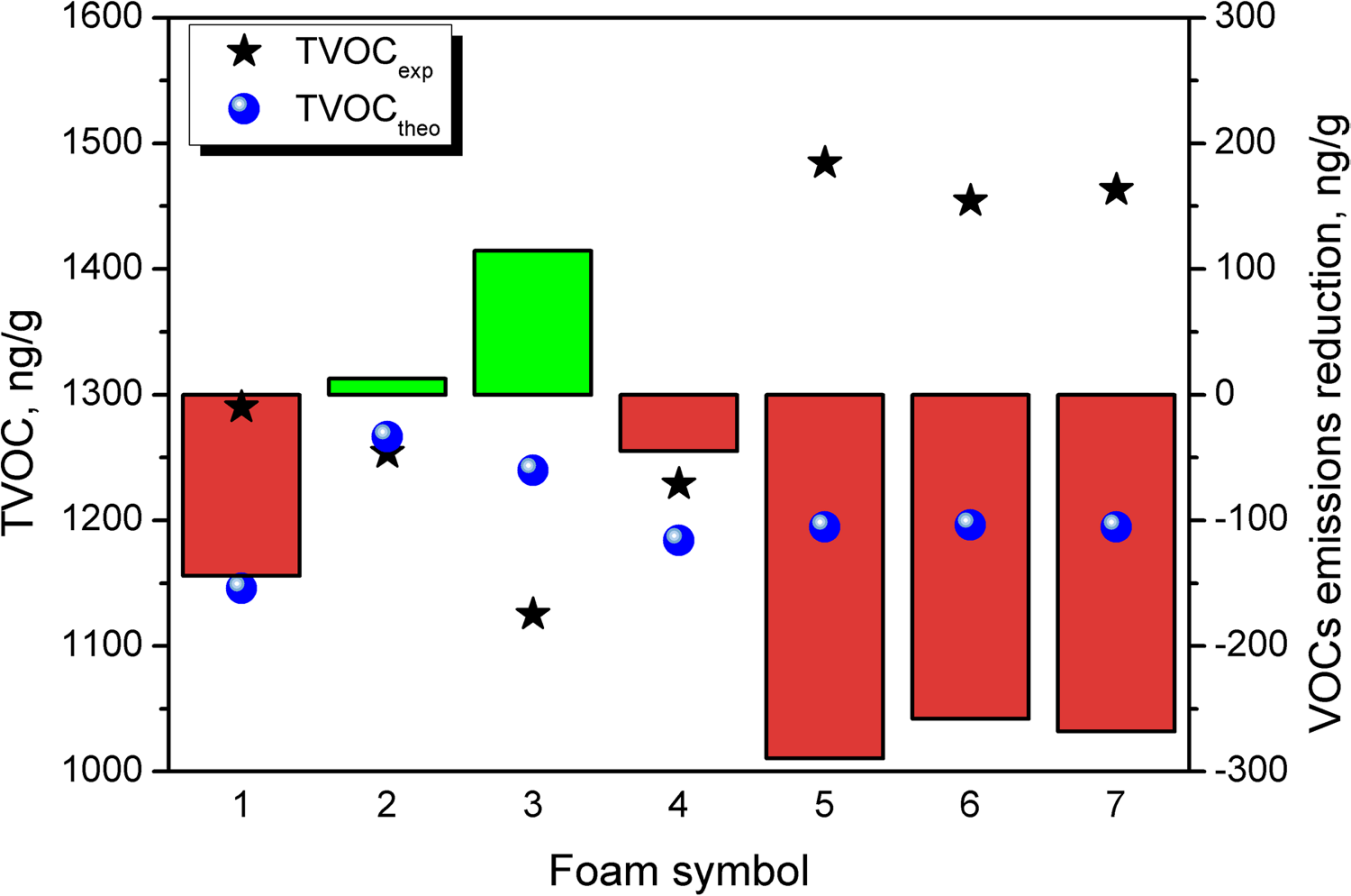
Theoretical and experimental TVOC values and calculated VOC emission reduction values for prepared PU/GTR composites

Such an effect was observed in the case of composites containing GTR/zinc borate compositions modified at 80 rpm, as presented in Fig. 8. It compares the experimentally determined values of TVOC parameter with theoretical ones providing information about a potential reduction in filler emissions after incorporation into PU matrix. Theoretical values were calculated using TVOC values for neat foam and particular filler samples according to Eq. (2):

2 $${\textrm{TVOC}}_{\textrm{theo}}={\varphi}_{\textrm{matrix}}\bullet {\textrm{TVOC}}_{\textrm{matrix}}+{\varphi}_{\textrm{filler}}\bullet {\textrm{TVOC}}_{\textrm{filler}}$$

where *φ* is the weight fraction, equals 0.8696 for matrix and 0.1304 for filler.

It can be seen that for samples 2 and 3, the experimental TVOC values were lower than theoretical, pointing to the hindered emissions, which, based on the structure of the GTR particles surface, could be associated with the enhanced compatibility of prepared composites. For other samples, TVOC_exp_ values were noticeably higher than TVOC_theo_. It suggests that the applied thermomechanical treatment enhanced VOC emissions from GTR/zinc borate compositions but, at the same time, did not provide a sufficient level of interfacial adhesion, which could effectively reduce emissions from final composites.

**Fig. 9:**
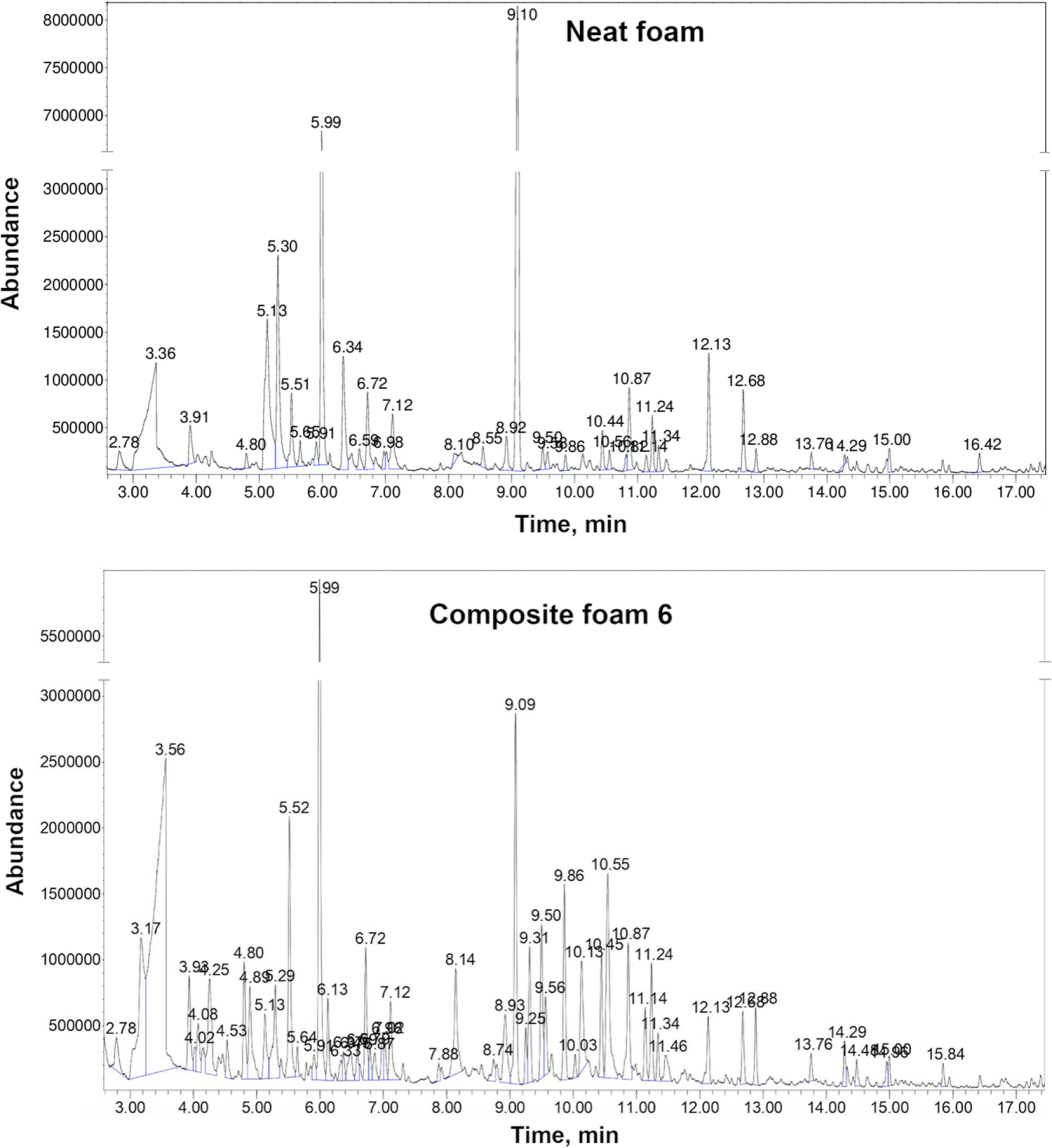
Chromatographs obtained during μCTE–TD–GC–MS analysis of neat foam and composite foam 6

Table 5 summarizes VOCs emitted from neat foam and composite foams filled with GTR/zinc borate compositions. Moreover, Fig. 9 presents the chromatographs obtained during μCTE–TD–GC–MS analysis of neat foam and composite 6, representing emissions detected from composite foams. The most noticeable peaks observed for 5.99, 9.10, and 12.13 minutes were attributed to the emissions of siloxane compounds from the sorption medium, similar to GTR/zinc borate compositions. Some contributions may also be attributed to the use of polyether polydimethylsiloxane surfactant in PU formulation. Oz et al. (Oz et al. 2019) reported the presence of octamethylcyclotetrasiloxane (noted at 9.10 min in the presented study) and decamethylcyclopentasiloxane (noted at 12.13 min) among the VOCs emitted from polyurethane mattresses, which was associated with the use of surfactants.

**Table 5:**
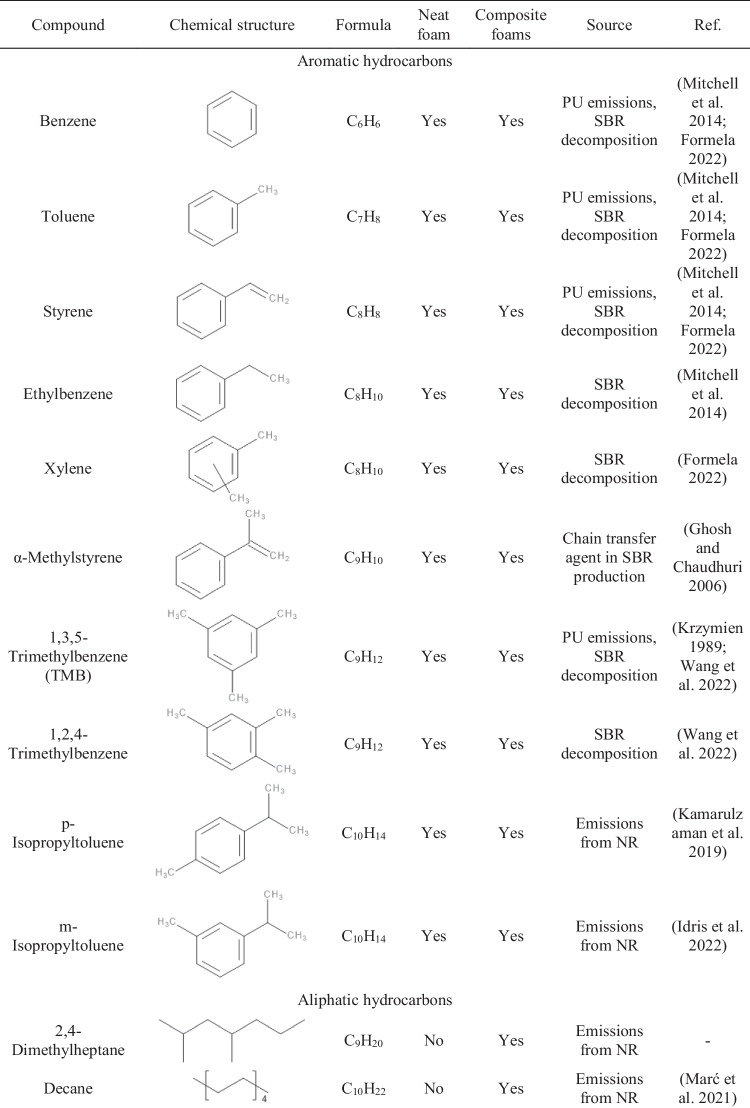

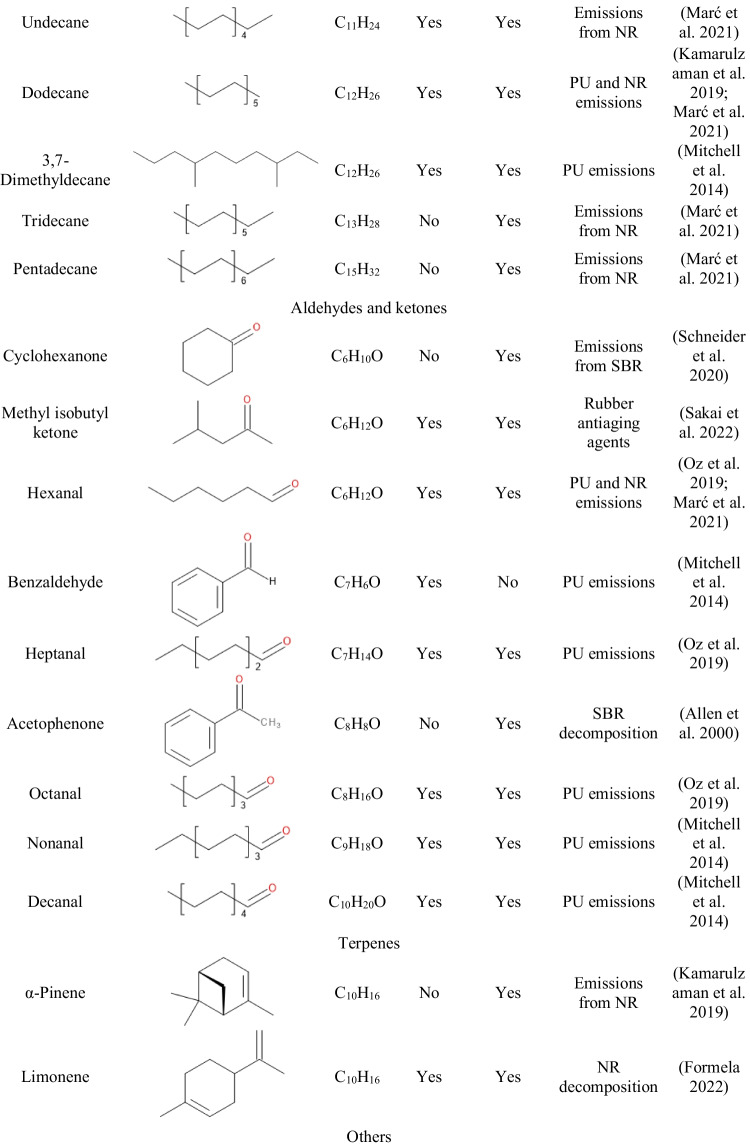

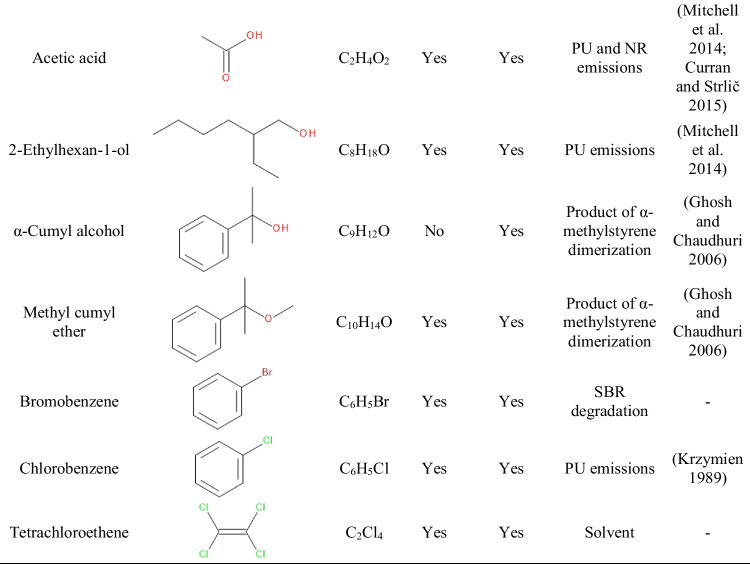
List of VOCs emitted from neat foam and PU/GTR composite foams along with their origin

For neat foam, the most abundant signals were noted for acetic acid, aromatics like toluene, chlorobenzene, or xylene, and aldehydes like hexanal, nonanal, or decanal. Acetic acid emissions have been reported by other researchers for different polyurethane materials, including coatings and foams (Tétreault et al. 1997; Mitchell et al. 2014). Various aromatics have been listed among the VOCs emitted from PU materials due to the common use of aromatic diisocyanates, e.g., toluene diisocyanate applied in the presented study, or the application of aromatic solvents (Oz et al. 2019). Emissions of aldehydes are attributed to the presence of catalysts applied in the PU industry and their oxidative degradation (Eling et al. 2020). Incorporation of GTR/zinc borate compositions resulted in additional signals originating from compounds detected during the analysis of fillers, like α-pinene, acetophenone, cyclohexanone, α-cumyl alcohol, or aliphatic hydrocarbons, whose presence can be ascribed to the decomposition of NR and SBR used during tire manufacturing as described above.

**Fig. 10:**
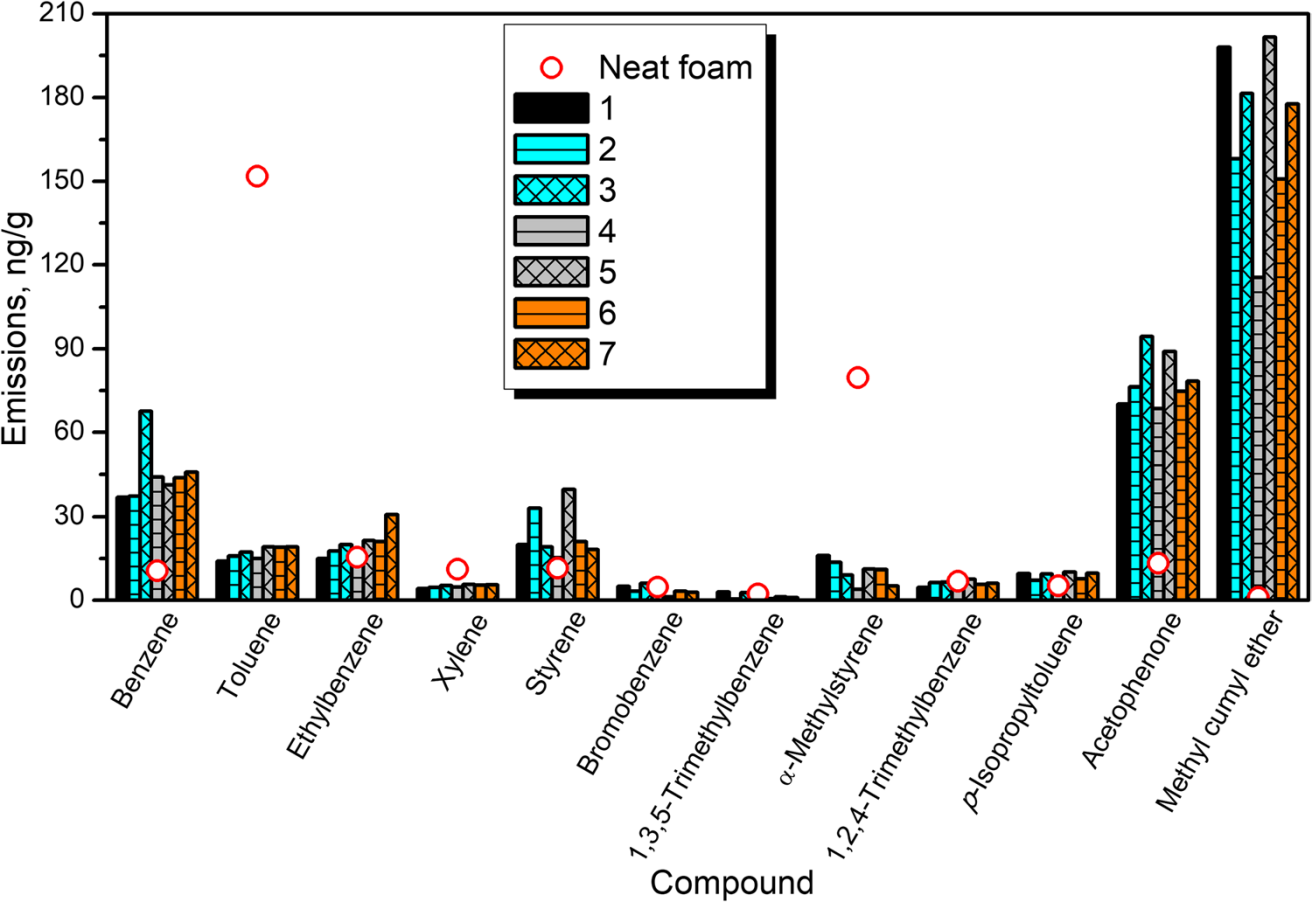
The detailed emissions of aromatic compounds from PU/GTR composites based on the applied certified reference material

Figure 10 provides insights into emissions of aromatics from PU/GTR composites and additionally prepared neat PU foam. It can be seen that the emission profile of unfilled PU foam is noticeably affected by the incorporation of GTR/zinc borate fillers. The most abundant aromatic compound emitted from unfilled foam is toluene, which has already been reported in previous works (Hillier et al. 2003; Oz et al. 2019). Such an effect can be attributed to the applied isocyanate—technical grade toluene diisocyanate, which can be a precursor for the toluene emissions (Oz et al. 2019). Reduction in its emissions from foam after incorporation of GTR/zinc borate compositions may be attributed to the presence of functional groups with an active hydrogen atom, mostly hydroxyls, on the surface of rubber particles. As presented in our previous papers (Kosmela et al. 2021; Olszewski et al. 2022b), GTR, especially after surface modifications, shows noticeable reactivity with isocyanates. These reactions occur to such an extent that they show a significant impact on the stoichiometry of polyaddition reactions leading to polyurethane formation (Olszewski et al. 2022b).

Another aromatic VOC, whose strong emission from neat foam has been reduced after filler incorporation, was α-methylstyrene. According to other works (Lattuati-Derieux et al. 2013; Tsochatzis et al. 2020), its emissions are typical for styrene-based polymers like polystyrene or acrylonitrile-butadiene-styrene copolymer. However, based on the presented data and considering the reduction in its emission similar to toluene, it can be suggested that the source of α-methylstyrene emission is also applied isocyanate. Nevertheless, direct confirmation requires further research in this area.

On the other hand, emissions of benzene, acetophenone, and methyl cumyl ether have been significantly enhanced after GTR/zinc borate incorporation into foamed PU matrix. Such an effect indicates that they originated from GTR and the decomposition of its components. The rest of the analyzed aromatics have been emitted at a similar level from neat foam and composites, suggesting that their emissions result from the chemical composition of both phases.

Table 6 provides values of PCC coefficients for the analyzed VOC emissions from prepared composites. The strongest correlation (bolded PCC values) can be observed for toluene and xylene, which have been reported as the two most abundant aromatic VOCs emitted from polyurethane foams (Marć et al. 2017). Both of these compounds also showed a strong correlation with ethylbenzene, another aromatic VOC reported to be emitted by PU foams (Plaisance et al. 2017). Considering the impact on the TVOC parameter, the highest PCC values were noted for toluene, 1,2,4-trimethylbenzene, and acetophenone, whose emissions were directly correlated with the chemical composition of PU matrix, as well as the decomposition of SBR during GTR/zinc borate thermomechanical treatment.

**Table 6:** Pearson correlation coefficients for emissions of aromatic compounds from prepared PU/GTR composites

	1	2	3	4	5	6	7	8	9	10	11	12	13
1: TVOC	1.00	0.17	**0.66**	0.38	0.52	0.57	0.15	**−**0.20	0.32	**0.75**	0.12	**0.64**	0.52
2: benzene		1.00	0.25	0.19	0.35	**−**0.35	0.53	0.46	**−**0.38	0.24	0.26	**0.69**	0.04
3: toluene			1.00	**0.76**	**0.97**	0.28	**−**0.29	**−**0.39	**−**0.28	0.71	0.31	0.55	0.24
4: ethylbenzene				1.00	**0.73**	**−**0.11	**−**0.28	**−**0.36	**−**0.59	0.37	0.36	0.25	0.10
5: xylene					1.00	0.24	**−**0.37	**−**0.39	**−**0.44	**0.73**	0.36	**0.60**	0.15
6: styrene						1.00	**−**0.41	**−**0.47	0.47	**0.75**	0.08	0.39	0.44
7: bromobenzene							1.00	**0.85**	0.38	**−**0.29	0.07	0.22	0.29
8: 1,3,5-TMB								1.00	0.32	**−**0.41	0.39	0.15	0.39
9: α-methylstyrene									1.00	**−**0.02	**−**0.04	**−**0.02	0.54
10: 1,2,4-TMB										1.00	0.32	**0.81**	0.42
11: p-isopropyltoluene											1.00	0.51	**0.77**
12: acetophenone												1.00	0.54
13: methyl cumyl ether													1.00

Except for the VOC emissions, thermomechanical treatment of GTR/zinc borate compositions affected their interactions with polyurethane matrix in prepared composites inducing changes in functional and mechanical performance, as well as another, one of the most important aspect, often crucial for final recipients—their appearance. Therefore, it is important to analyze the impact of introduced fillers on the appearance of composite material. Table 7 presents the impact of applied GTR/zinc borate fillers on the appearance of prepared PU composites in a quantitative and qualitative manner. It can be seen that the parameter affected most significantly was lightness (L*), which can be attributed to the incorporation of black GTR particles. The effect was especially pronounced for composites 2 and 3, which contained GTR/zinc borate compositions thermomechanically modified at 80 rpm. As mentioned above, such treatment resulted in the biggest decrease in GTR particle size, as well as the most significant surface development (Figs. 2, 3, and 4). As a result, smaller GTR particles were more evenly distributed in the foamed PU matrix resulting in the darkening of the PU phase and reduced contrast between colors of matrix and filler phases, which can be seen in images of samples’ cores presented in Table 7. Color parameters L*, a*, and b* were also used to calculate the total color difference parameter (ΔE*) with respect to the composite containing untreated GTR/zinc borate composition. It can be seen that for samples 2 and 3, the highest values of ΔE* were noted, pointing to the most significant color difference. According to the standard ISO 2813:2001, ΔE* parameter quantifies the human ability to distinguish color and notice the color difference (Hejna et al. 2021b). Values exceeding 5 indicate large color variations, which confirms the differences visualized in Table 7. For samples 4–7, values of ΔE* were noticeably lower, pointing to the small (1 < ΔE* < 2) or medium (2 < ΔE* < 3.5) variations (Bociaga and Trzaskalska 2016). Insignificant color differences are in line with only minor changes in GTR/zinc borate particle size and roughness resulting from thermomechanical treatment at higher values of screw speed. Except for the L* and ΔE*, other color parameters were hardly affected by the type of introduced GTR/zinc borate fillers. Such an effect can be explained by low values of a*, b*, and chroma characteristics for grey colors (Hejna et al. 2021a). Considering hue, its impact on the color is negligible for low chroma, which quantifies color saturation (Żukowska et al. 2022).

**Table 7:**
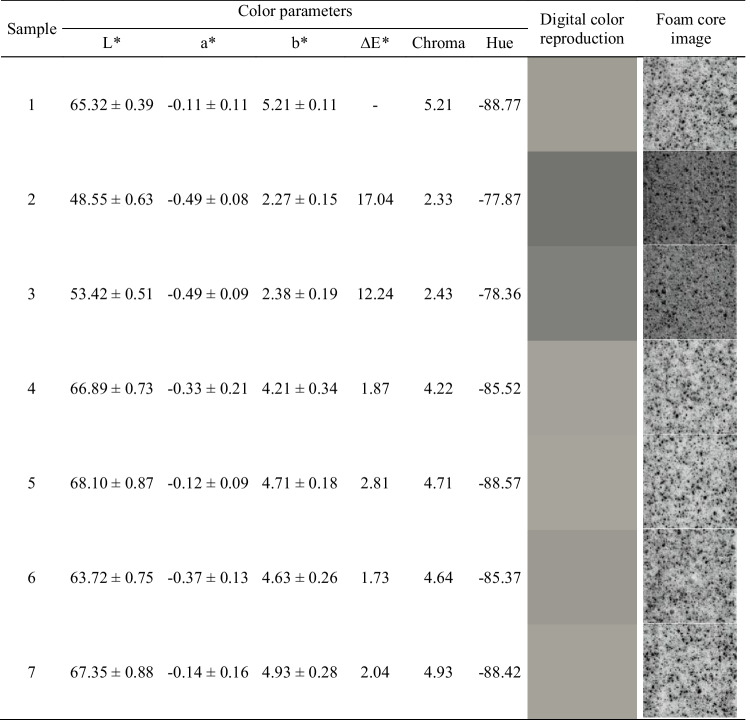
The impact of applied GTR/zinc borate fillers on color parameters and appearance of prepared composites

**Fig. 11:**
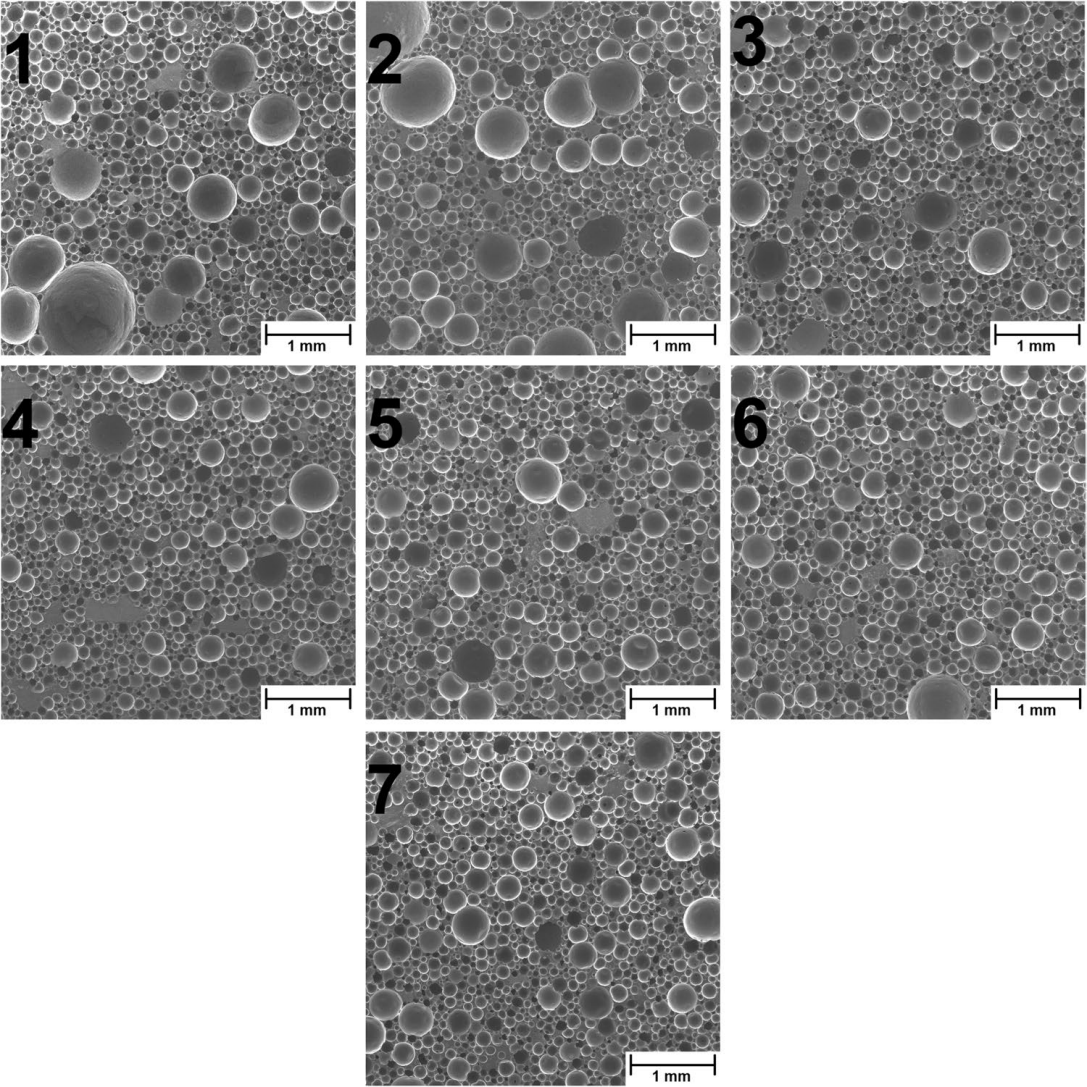
SEM images of prepared polyurethane composite foams (magnification of 50×)

Thermomechanical treatment of GTR/zinc borate compositions and resulting changes in the particle size distribution provided beneficial changes in the interfacial adhesion between fillers and PU matrix, which enhanced the cellular structure of composites. Such an effect was associated with the enhanced structure’s homogeneity and increased nucleating activity of treated GTR particles induced by their surface development, which decreased average particle size, as presented in Fig. 11 and Table 8.

**Table 8:** Parameters of cellular structure and thermal conductivity coefficient of prepared foams

Parameter	1	2	3	4	5	6	7
Average cell size (μm)	192 ± 116	186 ± 106	172 ± 98	166 ± 91	163 ± 86	160 ± 83	163 ± 90
Circularity	0.35 ± 0.21	0.36 ± 0.23	0.50 ± 0.27	0.51 ± 0.27	0.54 ± 0.27	0.54 ± 0.24	0.59 ± 0.27
Aspect ratio	1.34 ± 0.31	1.32 ± 0.24	1.32 ± 0.27	1.31 ± 0.22	1.29 ± 0.22	1.29 ± 0.19	1.29 ± 0.23
Roundness	0.78 ± 0.15	0.78 ± 0.12	0.78 ± 0.12	0.78 ± 0.12	0.80 ± 0.12	0.79 ± 0.11	0.79 ± 0.12
Open cell content (%)	57.66	57.07	54.54	54.31	53.15	56.13	59.29
*λ* coefficient (mW/(m·K))	69.15	66.65	65.56	68.21	64.97	68.63	69.16

It can be seen that despite the most significant changes in GTR/zinc borate compositions’ particle size distribution noted for the screw speed of 80 rpm, the changes between the cellular structures of samples 1–3 were not very significant. Such an effect was related to the compressing of zinc borate particles onto the surface of GTR, which apparently reduced the fillers’ nucleating activity despite the enhanced surface development indicated by Figs. 2, 3, and 4. Application of higher screw speeds during GTR modification showed a more beneficial impact on foams’ cellular structure, characterized by higher homogeneity and smaller average particle size. Such an effect points to the higher nucleating activity of fillers resulting from the presence of micrometric zinc borate particles and the roughened surface of GTR particles caused by the thermomechanical treatment. Independently of the applied GTR treatment conditions, composite foams were characterized with an open cell content in the range of 53.1–59.3% and thermal conductivity coefficient (*λ*) in the range of 64.9–69.2 mW/(m·K), which should satisfy the requirements for the floor underlays (Seo et al. 2016). Considering the thermal insulation performance of PU and other composite cellular materials, it benefits from interfacial compatibility (Cao et al. 2019; Zhu et al. 2020). Differences in the reduction of VOC emissions pointed to the varying interfacial compatibility of prepared composites, which also affected the thermal conductivity coefficient. The most notable inhibition of emissions was noted for samples 2 and 3, which are characterized by the noticeably lower *λ* value than composite containing GTR/zinc borate premix. Surprisingly, the lowest value of the thermal conductivity coefficient was noted for sample 5, which could be associated with the most beneficial combination of structural parameters—average cell size and open cell content. As repetitively reported in the literature (Hejna et al. 2018, 2021d; Andersons et al. 2020; Kosmela et al. 2021), the low values of these parameters are crucial for efficient thermal insulation performance, because they directly affect the extent of convective and radiative heat transfer.

Table 9 presents the results of performed static and dynamic mechanical tests. They point to the increase in foams’ tensile strength resulting from the incorporation of thermomechanically treated GTR/zinc borate compositions compared to the premix. The enhancement was noted irrespectively of the applied modification conditions meaning that all applied treatments caused noticeable enhancement of interfacial interactions resulting from the increased roughness of GTR particles’ surface compared to premix of GTR with zinc borate. Moreover, thermomechanical treatment in the twin-screw extruder was performed under an air atmosphere, which enables partial oxidation of ground tire rubber surface due to the combined actions of shear forces and oxygen present in the atmosphere. Our previous works dealt with GTR modification and noted such effects (Zedler et al. 2020b). Oxidation of the GTR surface may result in the generation of multiple functional groups, including hydroxyls, which can react with the isocyanate present in the reaction mixture (Gągol et al. 2015). As a result, the interfacial interactions between the polyurethane matrix and GTR may be noticeably enhanced due to the generation of covalent urethane bonds (Hejna et al. 2020).

**Table 9:** Results of static and dynamic mechanical tests performed for prepared composite foams

Parameter	1	2	3	4	5	6	7
Tensile strength (kPa)	701 ± 112	993 ± 104	1009 ± 93	974 ± 10	802 ± 19	1019 ± 81	915 ± 43
Elongation at break (%)	152 ± 42	156 ± 14	152 ± 10	163 ± 12	175 ± 6	160 ± 10	150 ± 3
Toughness (J/cm^3^)	71.8 ± 24.4	91.4 ± 18.4	92.1 ± 16.3	92.4 ± 2.3	76.6 ± 1.5	99.2 ± 21.5	79.6 ± 5.7
*E′* at 25 °C (MPa)	1.94	5.34	5.40	3.96	4.45	5.23	5.24
*T*_g_ of GTR (°C)	**−**49.2	**−**49.8	**−**48.3	**−**50.0	**−**49.3	**−**48.5	**−**48.6
*T*_g_ of PU foam (°C)	19.7	33.9	32.3	26.3	21.4	24.9	25.3

Dynamic mechanical analysis revealed the significant increase in composites’ stiffness expressed by the rise of storage modulus (*E*^′^), which was related to the enhanced interfacial interactions between matrix and filler. These interactions resulted from the above-mentioned chemical reactions between isocyanates present in the polyurethane formulation and hydroxyl groups generated on the GTR surface during thermomechanical treatment. Moreover, changes in the treatment parameters resulted in the glass transition temperature (*T*_g_) shifts, determined as the temperature position of peaks on the loss tangent curve. All samples showed two peaks attributed to the glass transitions of GTR (between **−**50.0 and **−**48.3 °C) and polyurethane (between 19.7 and 33.9 °C) (Saeb et al. 2022). The treatment hardly affected the *T*_g_ of GTR, indicating that the modification of particles occurred rather on their surface than in bulk.

On the other hand, the transition attributed to the PU matrix was significantly affected by the applied rubber treatment and its parameters. For polyurethane materials, *T*_g_ is very sensitive to the applied formulations and their modifications, such as filler incorporation, which may affect the desired balance between hydroxyl and isocyanate groups present in the system (Petrović et al. 2002). As mentioned above, changing the GTR treatment parameters may implicate variations in the content of functional groups on the surface of rubber particles, which may attract isocyanates. In the presented case, the mechanical performance of composite foams depended significantly on the applied GTR treatments due to the closeness of PU phase *T*_g_ and the ambient temperature of 22–23 °C, which were performed tensile tests. Therefore, it can be seen that the tensile strength and the stiffness of the material at 25 °C are increasing with the rise of T_g_.

**Fig. 12:**
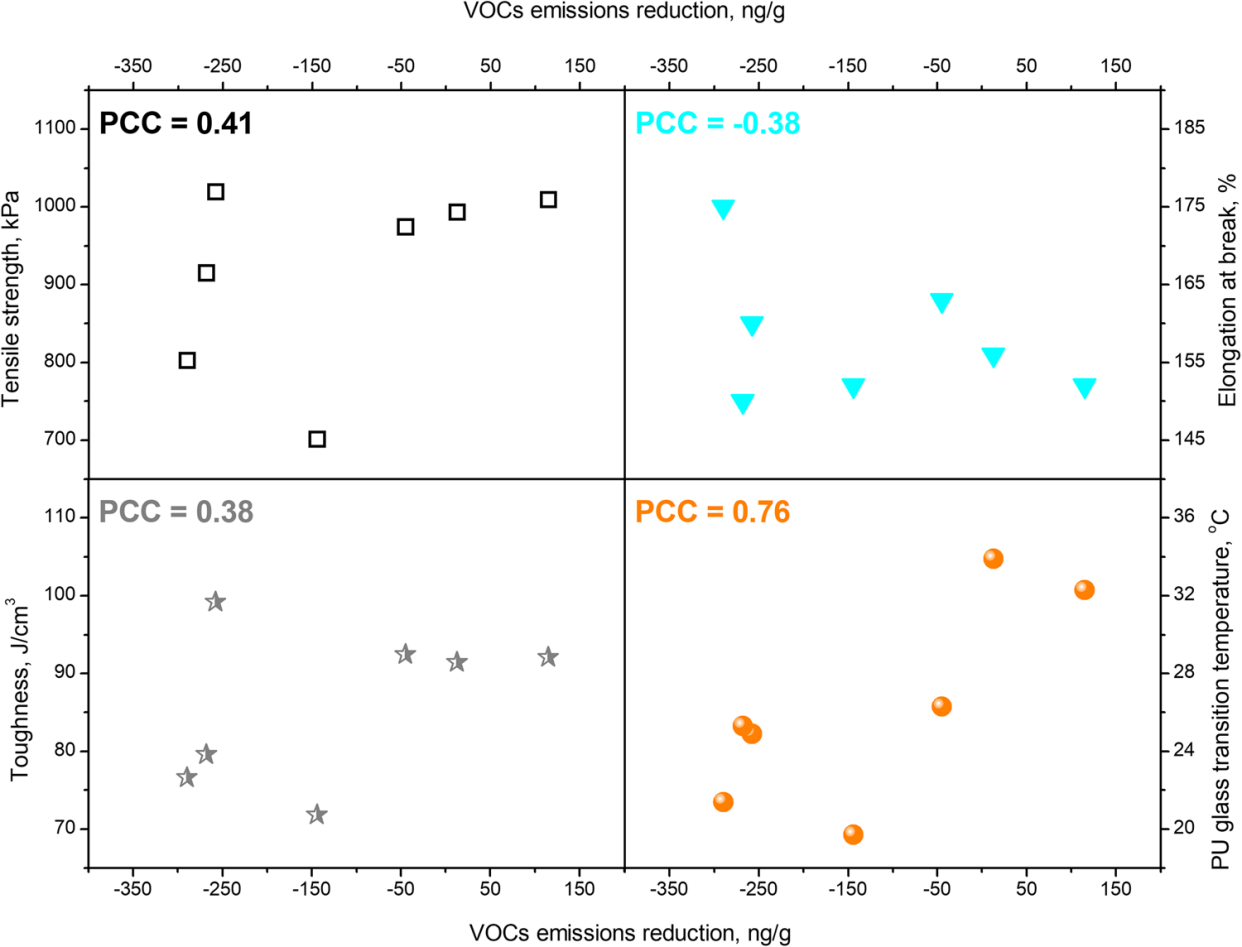
The impact of compatibility, quantified by the reduction in VOC emissions on the tensile performance and *T*_g_ of PU phase in prepared composites

The mechanical and thermomechanical performance of composite materials is also beneficent of good interfacial compatibility. To evaluate their dependence, Fig. 12 presents the impact of compatibility, quantified by the reduction in VOC emissions on the tensile performance of prepared composites, as well as the *T*_g_ of the PU phase, which, as mentioned above, is crucial for the mechanical performance. Moreover, values of Pearson correlation coefficients are provided, determining the power of the relationship between composite compatibility and their mechanical performance, as well as *T*_g_. Considering the tensile performance, especially tensile strength and toughness, it can be seen that they are increasing with the VOC emission reduction quantifying the strength of interfacial compatibility. However, due to the inconsistencies related to particular samples, the values of PCC point to a weak to medium correlation (Marć 2020). It indicates that the tensile performance of foamed composite materials is a very complex issue depending on many structural parameters.

On the other hand, a strong correlation can be observed between VOC emission reduction and *T*_g_ of the PU phase. Such an effect can be associated with the more direct impact of stoichiometric balance between hydroxyl and isocyanate groups during polyaddition on the glass transition of PU (Olszewski et al. 2022a). The surface development of GTR particles achieved during thermomechanical treatment is related to the increased surface roughness but also the generation of additional functional groups since treatment was conducted in an oxidative atmosphere. As a result, additional hydroxyl groups present on the surface of GTR particles may react with isocyanates and affect the stoichiometry of polyaddition, leading to PU generation (Olszewski et al. 2022b). As presented in Fig. 12, the strengthened compatibility shows a strong correlation with the increasing *T*_g_, which can be attributed to the covalent bonding at the interface enhancing the crosslink density of the PU phase.

Table 10 shows the impact of applied modifications of GTR/zinc borate compositions on the thermal decomposition behavior of prepared PU-based composite foams. Clearly, the thermomechanical treatment enabled a small enhancement of composite foams’ thermal stability, shifting the onset of decomposition by 4–7 °C towards higher temperatures. It is associated with the superior thermal stability of inorganic zinc borate compared to ground tire rubber and the additional friction during thermomechanical modification of GTR, which could enhance the specific surface area and reduce GTR particle size. These effects may beneficially influence the compatibility of presented composites. Moreover, shear forces acting on the GTR particles during thermomechanical treatment might cause their partial decomposition, resulting in higher thermal stability.

**Table 10:** Results of thermogravimetric analysis of prepared composite foams

Sample	*T*_-2%_ (°C)	*T*_-5%_ (°C)	*T*_-10%_ (°C)	*T*_-50%_ (°C)	*T*_max1_ (°C)	*T*_max2_ (°C)	Residue (wt%)
1	242.2	261.1	281.5	418.3	286.3	424.3	10.85
2	246.5	261.8	280.1	416.5	268.6	423.3	11.61
3	246.2	263.8	282.5	419.2	267.4	424.0	10.85
4	247.6	263.1	282.3	419.2	264.2	423.0	11.85
5	249.1	263.8	284.4	420.2	268.4	424.3	11.97
6	248.1	264.7	285.2	420.8	269.1	424.3	11.64
7	248.8	266.1	286.4	421.3	271.2	424.9	12.20

The incorporation of GTR waste into various polymers has attracted much interest, also due to the possibility of improving acoustic properties (Juliá Sanchis et al. 2012; Zhang et al. 2013; Maderuelo-Sanz et al. 2013; Colom et al. 2014). Juliá Sanchis et al. (Juliá Sanchis et al. 2012) investigated the effect of GTR granulometry and GTR-fiber systems on the properties of multilayer panels. Results of investigations showed that using GTR alone does not allow obtaining acoustic improvement, while in a complex multiphase system it shows good sound absorptions. Also, as demonstrated by Colom et al. (Colom et al. 2014), the incorporation of unmodified GTR into recycled PVC did not allow the improvement of the acoustic properties. On the other hand, beneficial effects can be obtained through the simultaneous presence of GTR and adjustment of the composite’s cellular structure. It suggests that obtaining favorable acoustic properties will not be related only to the presence of the waste elastomer filler itself because it is a complex issue including the final product’s structure, i.e., its porosity, tortuosity, thickness, and damping material arrangement (Maderuelo-Sanz et al. 2013). Therefore, GTR application in PU foams is reasonable and necessary to verify, especially considering applied thermomechanical treatments and resulting variations in PU cellular structure.

**Fig. 13:**
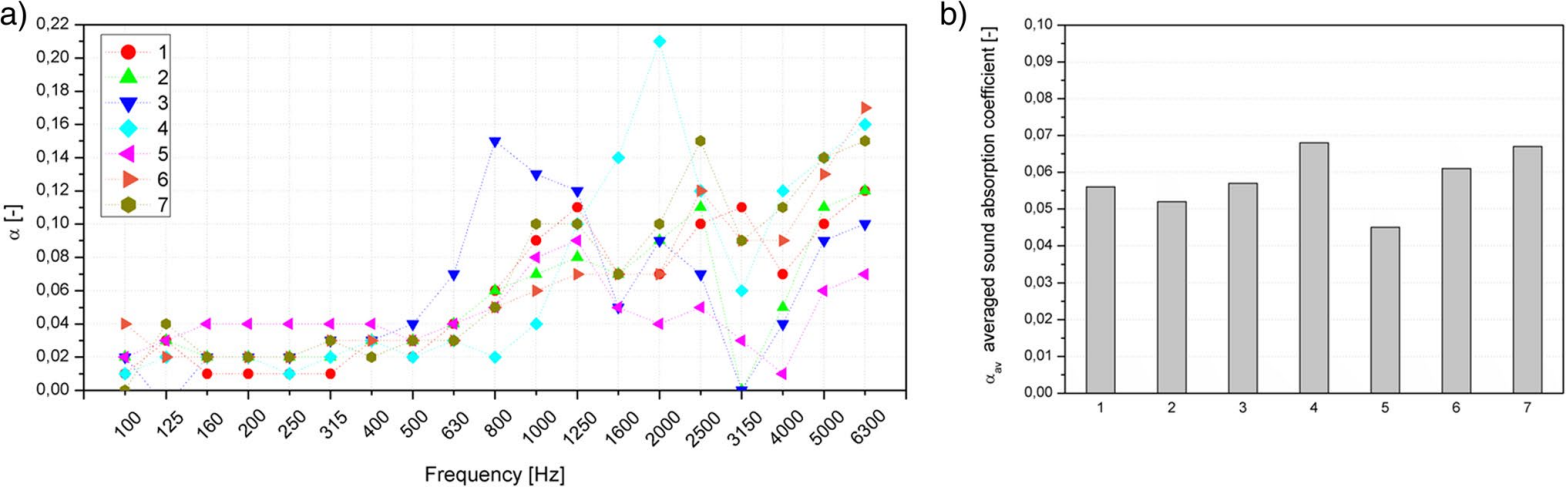
Sound absorption coefficients of polyurethane foams (**a**) and average sound absorption coefficient (**b**)

The acoustic properties of cellular materials, including PU foams, significantly depend on the structure of the pores. It is assumed that the increase in the effectiveness of sound suppression is related to the number of open pores; moreover, it has been shown that beneficial effects are obtained for foams with larger pore diameters. This effect is related to airflow in the internal structure of the foam and the conversion of the sound energy into heat by the attenuation of the cell walls’ micro deformations (Zhang et al. 2012). Figure 13 summarizes the sound absorption coefficients as a frequency (a) function and the averaged sound absorption coefficient (b). The data obtained for all material series (Fig. 13a) are repeatable. The shape of the obtained characteristics and the maximum values recorded in the selected frequency bands are relatively low. All samples showed low *α* values at low frequencies (below 500 Hz). In the considered case, as shown by the analysis of the foams’ structure, all series can be classified as microporous. Moreover, taking into account the standard deviations, there is no significant variation in the size of the pores between the series. The analysis of the open cell content made it possible to observe that only in the case of samples 1, 2, and 7, their content was increased. Considering the *α*_avg_, samples 4 and 7 showed the best suppressing effects, but there is no clear correlation between acoustic properties and structure. While series 7 had the highest content of open pores, series 4 did not show any structural properties or reduced elasticity, which could increase the material’s sound absorption. To conclude, all the calculated values of the average sound attenuation coefficient are low (<0.1), which does not allow the tested foams to be classified as materials that can be used as soundproofing materials.

## Conclusions

The presented study analyzed the thermomechanical modification of GTR with zinc borate as a potential GTR management technique aimed at its application as a filler in a flexible, foamed PU matrix. The GTR/zinc borate compositions were modified in the twin-screw extruder under varying temperatures and screw speeds and further introduced into flexible PU foams. The variations in GTR particle size and surface development were assessed as essential parameters for the performance of composite materials and correlated with the emissions of volatile organic compounds from waste material, which is commonly considered burdensome.

Obtained results revealed that thermomechanical modification, especially at low screw speed, enabled a noticeable reduction of the GTR particle size and substantial surface development, implicating intensified emissions of VOCs, including compounds potentially harmful to human health and the environment. On the other hand, applying GTR/zinc borate compositions characterized by the highest surface development and VOC emissions as fillers enabled the most substantial hindering of emissions compared to expected results and was accompanied by the most beneficial cellular structure, thermal insulation performance, and mechanical properties. Such an effect was attributed to the contradictory impact of thermomechanical treatment at low screw speed related to the simultaneous facilitating of VOC emissions from filler and providing additional interactions with PU matrix, including covalent bonding, leading to strengthening of interfacial adhesion, better encapsulation of GTR particles by PU matrix, and hindering of VOC emissions from final composite. Changes at the interface were mirrored in the shifts of the glass transition temperature of the PU matrix, affecting the molecular motions determining the strength of the material. Therefore, such modifications should be considered beneficial considering GTR application in composite materials.

To summarize, the thermomechanical treatment of GTR in a twin-screw extruder assisted by the presence of zinc borate and subsequent application of obtained compositions in flexible PU foams poses an auspicious waste tire method utilization. The adjustment of treatment conditions enables the manufacturing of composite materials with a satisfactory mechanical and thermal performance characterized by reduced VOC emissions, hindering the hazardous environmental impacts of GTR. As a result, prepared materials could potentially be applied in the construction and building sector, e.g., floor underlays, due to the beneficial mechanical and thermal insulation performance.

### Supplementary Information

ESM 1: Supplementary Figure 1 (JPG 901 kb)

ESM 2: (DOCX 37 kb)

## Data Availability

The data were produced as a result of studies carried out in the university laboratory. Those who want to get information should contact the corresponding author.
