# Supplementary figures and images for: Management of ground tire rubber waste by incorporation into polyurethane-based composite foams
Source: Environ Sci Pollut Res Int. 2023 Jan 26;31(12):17591–616. doi: 10.1007/s11356-023-25387-w (PMC10923751; doi:10.1007/s11356-023-25387-w)

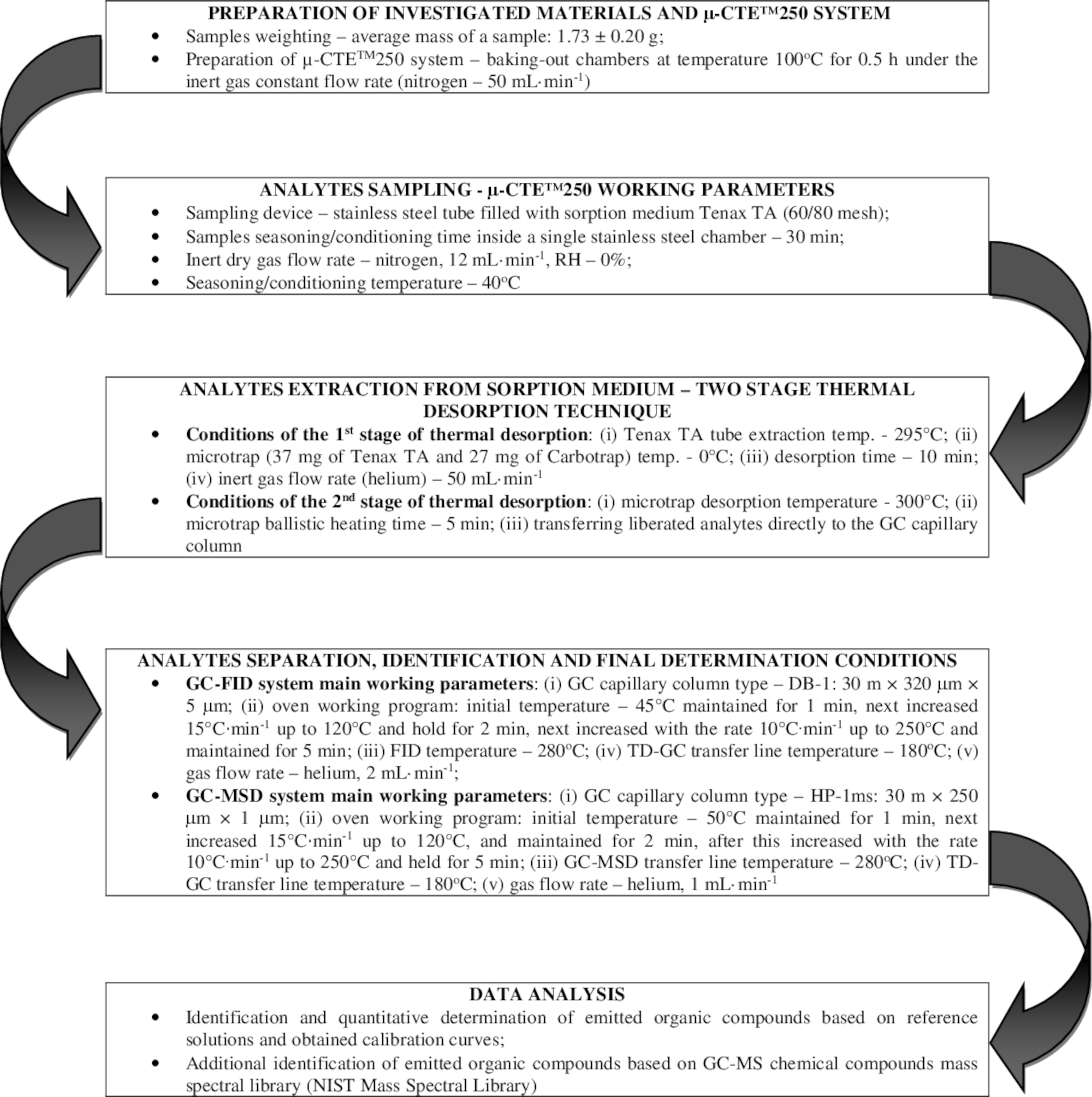

Supplement: Supplementary file 1 — Supplementary Figure 1 (JPG 901 kb) [file 11356_2023_25387_MOESM1_ESM.jpg]
