# Supplementary material for: Management of ground tire rubber waste by incorporation into polyurethane-based composite foams
Source: Environ Sci Pollut Res Int. 2023 Jan 26;31(12):17591–616. doi: 10.1007/s11356-023-25387-w (PMC10923751; doi:10.1007/s11356-023-25387-w)
Supplement: Supplementary file 2 — (DOCX 37 kb) [file 11356_2023_25387_MOESM2_ESM.docx]

| **PREPARATION OF INVESTIGATED MATERIALS AND μ-CTE™250 SYSTEM**   - Samples weighting – average mass of a sample: 1.73 ± 0.20 g; - Preparation of µ-CTE^TM^250 system – baking-out chambers at temperature 100^o^C for 0.5 h under the inert gas constant flow rate (nitrogen – 50 mL·min^-1^) |
| --- |
|  |
| **ANALYTES SAMPLING - μ-CTE™250 WORKING PARAMETERS**   - Sampling device – stainless steel tube filled with sorption medium Tenax TA (60/80 mesh); - Samples seasoning/conditioning time inside a single stainless steel chamber – 30 min; - Inert dry gas flow rate – nitrogen, 12 mL·min^-1^, RH – 0%; - Seasoning/conditioning temperature – 40^o^C |
|  |
| **ANALYTES EXTRACTION FROM SORPTION MEDIUM – TWO STAGE THERMAL DESORPTION TECHNIQUE**   - **Conditions of the 1^st^ stage of thermal desorption**: (i) Tenax TA tube extraction temp. - 295°C; (ii) microtrap (37 mg of Tenax TA and 27 mg of Carbotrap) temp. - 0°C; (iii) desorption time – 10 min; (iv) inert gas flow rate (helium) – 50 mL·min^-1^ - **Conditions of the 2^nd^ stage of thermal desorption**: (i) microtrap desorption temperature - 300°C; (ii) microtrap ballistic heating time – 5 min; (iii) transferring liberated analytes directly to the GC capillary column |
|  |
| **ANALYTES SEPARATION, IDENTIFICATION AND FINAL DETERMINATION CONDITIONS**   - **GC-FID system main working parameters**: (i) GC capillary column type – DB-1: 30 m × 320 μm × 5 μm; (ii) oven working program: initial temperature – 45°C maintained for 1 min, next increased 15°C⋅min^-1^ up to 120°C and hold for 2 min, next increased with the rate 10°C∙min^-1^ up to 250°C and maintained for 5 min; (iii) FID temperature – 280^o^C; (iv) TD-GC transfer line temperature – 180^o^C; (v) gas flow rate – helium, 2 mL·min^-1^; - **GC-MSD system main working parameters**: (i) GC capillary column type – HP-1ms: 30 m × 250 μm × 1 μm; (ii) oven working program: initial temperature – 50°C maintained for 1 min, next increased 15°C⋅min^-1^ up to 120°C, and maintained for 2 min, after this increased with the rate 10°C∙min^-1^ up to 250°C and held for 5 min; (iii) GC-MSD transfer line temperature – 280^o^C; (iv) TD-GC transfer line temperature – 180^o^C; (v) gas flow rate – helium, 1 mL·min^-1^ |
|  |
| **DATA ANALYSIS**   - Identification and quantitative determination of emitted organic compounds based on reference solutions and obtained calibration curves; - Additional identification of emitted organic compounds based on GC-MS chemical compounds mass spectral library (NIST Mass Spectral Library) |
